# Neuronal development is promoted by weakened intrinsic antioxidant defences due to epigenetic repression of Nrf2

**DOI:** 10.1038/ncomms8066

**Published:** 2015-05-13

**Authors:** Karen F.S. Bell, Bashayer Al-Mubarak, Marc-André Martel, Sean McKay, Nicola Wheelan, Philip Hasel, Nóra M. Márkus, Paul Baxter, Ruth F. Deighton, Andrea Serio, Bilada Bilican, Sudhir Chowdhry, Paul J. Meakin, Michael L.J. Ashford, David J.A. Wyllie, Robert H. Scannevin, Siddharthan Chandran, John D. Hayes, Giles E. Hardingham

**Affiliations:** 1 Centre for Integrative Physiology, University of Edinburgh, Edinburgh EH8 9XD, UK; 2 MRC Centre for Regenerative Medicine, University of Edinburgh, Edinburgh EH16 4SB, UK; 3 Medical Research Institute, University of Dundee, Ninewells Hospital and Medical School, Dundee DD1 9SY, UK; 4 Biogen Idec, 14 Cambridge Center, Cambridge, Massachusetts 02142, USA

## Abstract

Forebrain neurons have weak intrinsic antioxidant defences compared with astrocytes, but the molecular basis and purpose of this is poorly understood. We show that early in mouse cortical neuronal development *in vitro* and *in vivo*, expression of the master-regulator of antioxidant genes, transcription factor NF-E2-related-factor-2 (Nrf2), is repressed by epigenetic inactivation of its promoter. Consequently, in contrast to astrocytes or young neurons, maturing neurons possess negligible Nrf2-dependent antioxidant defences, and exhibit no transcriptional responses to Nrf2 activators, or to ablation of Nrf2’s inhibitor Keap1. Neuronal Nrf2 inactivation seems to be required for proper development: in maturing neurons, ectopic Nrf2 expression inhibits neurite outgrowth and aborization, and electrophysiological maturation, including synaptogenesis. These defects arise because Nrf2 activity buffers neuronal redox status, inhibiting maturation processes dependent on redox-sensitive JNK and Wnt pathways. Thus, developmental epigenetic Nrf2 repression weakens neuronal antioxidant defences but is necessary to create an environment that supports neuronal development.

## Abstract (web summary)

Neurons in the brain are more susceptible to oxidative stress than astroglial cells but the molecular basis and biological reasons for this are poorly understood. Here the authors show that developing cortical neurons have reduced levels of the antioxidant transcription factor Nrf2 due to epigenetic silencing and that this is necessary for proper neuronal development.

## Graphical abstract

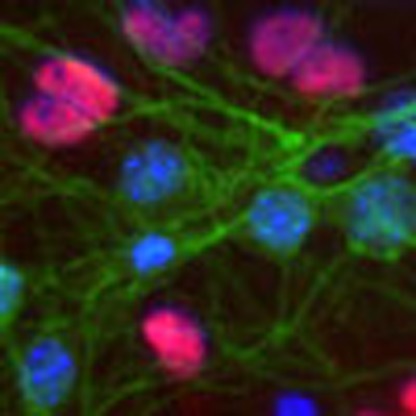

In isolation, neurons tend to be more vulnerable to oxidative insults than astrocytes partly as a result of weaker antioxidant defences resulting from lower levels of glutathione and reduced catalase activity^1^,^2^. Astrocytic-derived extrinsic support is known to play an important role in protecting neurons against excessive reactive oxygen species (ROS) production^3^,^4^,^5^, which is likely to be an important factor in maintaining neuronal viability throughout an organisms lifetime. However, the molecular mechanisms underlying these cell-type-specific differences in antioxidant defences are unclear. Understanding the basis for the low intrinsic antioxidant capacity of neurons may provide therapeutic targets aimed at boosting their antioxidant defences as a protective strategy against acute and chronic neurological and neurodegenerative disorders associated with oxidative stress^6^.

The biological rationale for the relatively weak intrinsic antioxidant defences of neurons is unknown. At face value, it seems paradoxical that neurons should be endowed with relatively weak antioxidant defences: not only do they generate more ROS than most cell types because of high metabolic activity, but also their post-mitotic and non-regenerative nature means that they must survive for many years without being rendered dysfunctional by accumulating oxidative damage. Forebrain neurons receive significant antioxidant support from surrounding astrocytes^4^,^5^ (for example, via supply of precursors for glutathione biosynthesis^7^), which is then utilized by neuronal systems^8^, however, the reason why neurons have evolved to need non-cell autonomous protection is unclear.

The transcription factor NF-E2-related factor 2 (Nrf2) is a master regulator of antioxidant defences that controls a battery of antioxidant and detoxification genes containing cognate-binding sites within their promoters (referred to as antioxidant response elements, AREs)^9^. Nrf2 is widely thought to be ubiquitously expressed and has been shown to protect cells against a variety of stress-induced diseases, and become dysregulated in certain cancers^10^,^11^. Basal levels of nuclear Nrf2 are normally quite low, as it is targeted for ubiquitin-mediated degradation by its inhibitor Keap1 in the cytoplasm. However, in response to oxidative stress or small-molecule inhibitors of Keap1, Nrf2 accumulates and translocates to the nucleus where it activates a battery of antioxidant and detoxification genes^11^,^12^. Examples of Nrf2 target genes include Catalase (*Cat*) and glutamate-cysteine-ligase catalytic subunit (*Gclc*), which catalyses the rate-limiting step of glutathione biosynthesis^13^,^14^,^15^. Activation of Nrf2-mediated gene expression in astrocytes, either via overexpression or pharmacological activation, confers non-cell autonomous protection to surrounding neurons in a variety of models, including rodent models of neurodegenerative disease, as well as human stem cell-derived neuron-astrocyte systems^7^,^16^,^17^. However, the neuronal Nrf2 pathway has been reported to be weak^4^,^5^, although how and why this is the case, is not well understood.

We show here that both basal and inducible Nrf2 activity in cortical neurons is extremely low due to epigenetic repression of the *Nrf2* gene promoter early in development. Moreover, we show that inactivation of the Nrf2 pathway provides a more flexible redox environment, which is crucial for key redox-sensitive signalling pathways that mediate early neuronal development. Collectively, our study provides both the molecular mechanism and the biological reason behind the apparent paradox of weak neuronal intrinsic antioxidant defences.

## Results

### Limited role for Nrf2 in neuronal antioxidant defenses

**Figure 1:**
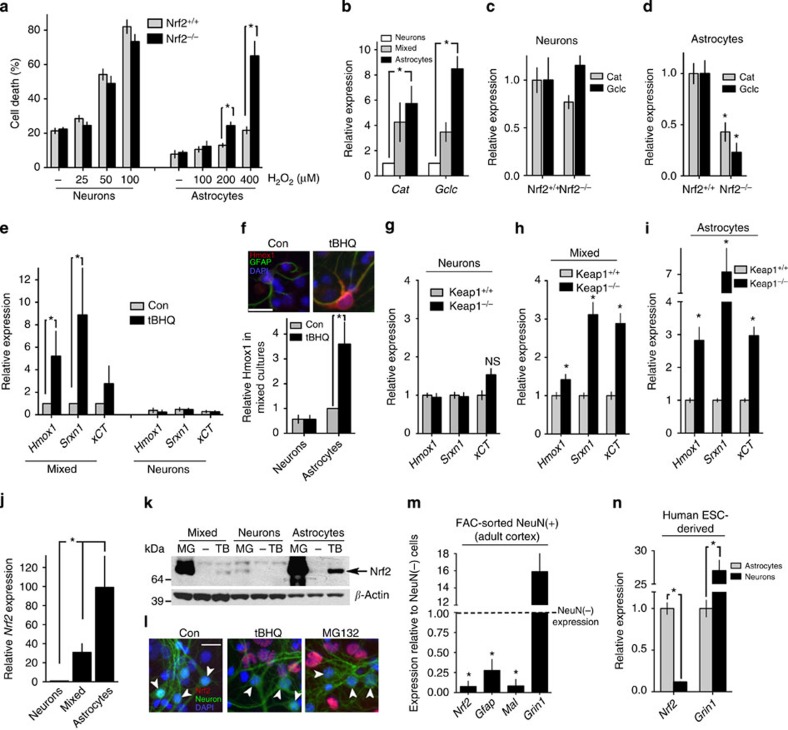
The Nrf2 pathway is weak in cortical neurons. (**a**) Impact of Nrf2 deficiency on vulnerability to H_2_O_2_ treatment, in DIV10 astrocytes (>98% GFAP^+^ astrocytes) or neurons (cortical cultures containing >98% NeuN^+^ neurons, <0.02% GFAP^+^ astrocytes). Cells were treated with H_2_O_2_, fixed after 24 h, DAPI stained and scored for survival/death based on nuclear morphology. *P<0.05, Student’s *t*-test here and throughout unless otherwise stated (neurons: *n*=4, 700–2000 cells analysed per condition per genotype (PCPG); astrocytes wt *n*=5, 700–2,000 cells PCPG, ko *n*=7, 2,100–2,700 cells PCPG). Data are displayed as mean±s.e.m. throughout. (**b**) Nrf2 target genes *Cat* and *Gclc* expression analysed by qRT–PCR, normalized to 18s rRNA, expressed relative to the level in pure neuronal cultures. Mixed cultures: approximately 10% GFAP^+^ astrocytes, 90% NeuN^+^ neurons. *P<0.05, *Cat n*=6, *Gclc n*=7. (**c**,**d**) Impact of Nrf2 deficiency on *Cat* or *Gclc* mRNA expression in neurons (**c**) and astrocytes (**d**). *P<0.05 compared with wild type (WT); *Cat n*=4, *Gclc n*=5. (**e**) qRT–PCR analysis of Nrf2 target gene (Hmox1, Srxn1 and xCT) expression in response to tBHQ (10 μM, 8 h) treatment of neuronal or mixed cultures. *P<0.05 (*n*=5–6). (**f**) Immunohistochemical analysis of Hmox1 expression in tBHQ-treated mixed cultures. *P<0.05 (*n*=5). Upper: example pictures illustrating elevation of Hmox1 in astrocytes within tBHQ-treated mixed cultures. Scale bar, 25 μm. (**g**–**i**) The effect of Keap1 deficiency on Nrf2 target genes. *P<0.05 (compared with WT control (Con), *n*=8–15). (**j**) *Nrf2* mRNA analysed in parallel cultures of the indicated types. *P<0.05, (*n*=10). (**k**) Western blot analysis of Nrf2 expression in different cell types treated with tBHQ (TB; 10 μM) or MG132 (MG; 5 μM) for 16 h. (**l**) Images of mixed cultures treated as indicated and stained with an anti-Nrf2 antibody (red) and a neuronal marker (Milli-Mark). White arrows highlight lack of Nrf2 induction in neurons. Scale bar, 25 μm. (**m**) qRT–PCR analysis of Nrf2 and neuronal/non-neuronal marker gene expression in RNA isolated from FAC-sorted adult cortical NeuN^+^ neurons, expressed relative to levels in NeuN^−^ cells. *P<0.05 (*n*=4). See Supplementary Fig. 1e. (**n**) Expression of *NRF2* (*NEF2L2*) and *GRIN1* analysed in astrocytes and neurons derived from H9 hESCs. *P<0.05 (*n*=3). NS, not significant.

As forebrain neurons have been reported to support lower basal and induced Nrf2-dependent gene expression than astrocytes^4^,^5^, we hypothesized that this may contribute to their differential vulnerability to oxidative stress. We observed that cortical neurons were more sensitive to an oxidative insult (H_2_O_2_) than astrocytes but that, unlike astrocytes, Nrf2 deficiency did not alter their vulnerability (Fig. 1a). Moreover, although neuronal expression of *Cat* and *Gclc*, key antioxidant Nrf2 target genes, was lower than in astrocytes (Fig. 1b), Nrf2 deficiency did not affect their levels in neurons (Fig. 1c), but did in astrocytes (Fig. 1d). Thus, in cortical neurons, basal Nrf2 activity does not contribute to intrinsic antioxidant defences, partly explaining why neuronal defences are weak compared with astrocytes.

### Hypo-expression of Nrf2 in cortical neurons

Neither pharmacological activation of Nrf2 by *tert*-butyl hydroquinone (tBHQ), nor genetic activation of Nrf2 by Keap1-knockout, triggered induction of the Nrf2 target genes *Hmox1*, *Srxn1* and *xCT* in neurons (Fig. 1e–g). In contrast, astrocytes, either on their own or in a mixed culture preparation (containing 90% neurons and 10% astrocytes) supported robust pharmacological activation (Fig. 1e,f) that was abolished in Nrf2^–/–^ cultures (Supplementary Fig. 1a for *Hmox1*^18^, for *xCT* and *Srxn1*), as well as elevated Nrf2 target gene expression in a Keap1-deficient background (Fig. 1h,i). We also performed experiments under 5% O_2_ conditions to eliminate the possibility that the neuronal Nrf2 pathway was maximally induced by ambient air conditions. However, unlike mixed astrocyte/neuronal cultures, neuronal cultures showed no induction of Nrf2 target genes by tBHQ at 5% O_2_ (Fig. S1b).

Cortical neurons are therefore unable to mount significant Nrf2-mediated transcriptional responses, raising the question about the mechanistic basis for this. Although Nrf2 protein is dynamically regulated at the level of Keap1-mediated degradation, the fact that no gene induction was observed in Keap1^–/–^ neurons suggests the lack of a functional Nrf2 pathway is due to pre-translational events. This concept is supported by our observations that forced expression of Nrf2 in neurons induces expression of Nrf2 target genes such as Srxn1 (ref. 19) and Hmox1 (Supplementary Fig. 1c), resulting in the protection of neurons against oxidative stress^19^ and oxygen-glucose deprivation (Supplementary Fig. 1d). This suggests that Nrf2 target genes are amenable to activation in neurons when Nrf2 is present, but that levels of endogenous neuronal Nrf2 are insufficient to mediate such induction. We therefore studied neuronal *Nrf2* mRNA expression and found it to be less than 2% of that found in astrocytes (Fig. 1j) consistent with previous observations^4^,^5^. Moreover, the increase in Nrf2 protein stimulated in neurons by tBHQ or proteosomal inhibition is markedly lower than in astrocytes or mixed neuronal-astrocyte cultures (Fig. 1k,l).

We wanted to determine whether hypo-expression of Nrf2 is also a feature of neurons *in vivo*. Adult mouse cortices were subjected to fluorescence-activated cell sorting (FACS), using a method that separates neurons from non-neurons based on NeuN-positive immunofluorescence, while preserving the capacity to extract RNA^20^ (Supplementary Fig. 1e). As well as predictable positive and negative enrichment for neuronal and non-neuronal markers, NeuN^+^ cells were found to express very low levels of Nrf2 (Fig. 1m), consistent with our *in vitro* observations. We also investigated whether neuronal Nrf2 hypo-expression is relevant to the human. Cortical-patterned neurons and astrocytes were generated from the same human embryonic stem cell (hESC) line (H9) as described previously^17^,^21^,^22^. Strikingly, hESC-derived neurons expressed far less Nrf2 than hESC-derived astrocytes (Fig. 1n, for comparison, *GRIN1* showed the reverse enrichment). Thus, *in vitro, in vivo* and in a model human system, Nrf2 is severely underexpressed in neurons as compared with astrocytes.

### Epigenetic repression of Nrf2 in developing neurons

**Figure 2:**
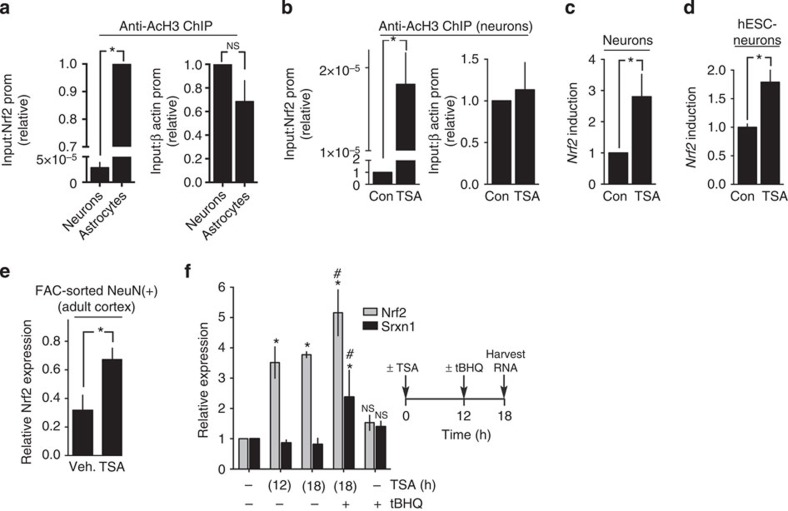
The Nrf2 promoter is epigenetically repressed in cortical neurons. (**a**) ChIP analysis of acetylated histone H3 (Ac-H3) occupancy at the Nrf2 (left) and β-actin (right) promoters in neurons and astrocytes, normalized to input and expressed relative to each other. *P<0.05, NS=no significant difference (*n*=5). (**b**) Effect of TSA treatment (8 h) on Ac-H3 levels at the Nrf2 and β-actin promoters in neurons. *P<0.05 (*n*=5). (**c**,**d**) Effect of TSA treatment on *Nrf2* expression in mouse cortical neurons (**c**) and human H9 ESC-derived neurons (**d**), normalized to Rpl13a. *P<0.05 (*n*=3). (**e**) Adult mice were subjected to intra-peritoneal injection of TSA (10 mg kg^−1^) or vehicle (Veh.) and at 8 h culled and cortical neurons obtained by enzymic dissociation of the cortex followed by FAC-sorting of NeuN^+^ cells. RNA was extracted immediately and Nrf2 expression studied. *P<0.05, (*n*=7 (Veh.), 4 (TSA)). (**f**) Pre-treating cultured cortical neurons with TSA renders the neuronal Nrf2 pathway amenable to activation by tBHQ. Neurons were treated as indicated in the schematic, and expression of Nrf2 and Nrf2 target gene Srxn1 analysed by qRT–PCR. (*P*<0.05 analysis of variance plus Tukey’s *post-hoc* test, *significant difference compared with control untreated cells; ^#^significant effect of tBHQ+TSA compared with TSA alone condition). ‘NS’ emphasizes non-significant effect of tBHQ versus untreated neurons (*n*=4 except control (no TSA, no tBHQ, *n*=5)).

We hypothesized that the hypo-expression of Nrf2 in neurons could be a result of epigenetic repression. Chromatin immunoprecipitation (ChIP) analysis of acetylated histone H3 associated with the Nrf2 promoter revealed a strong degree of hypo-acetylation in neurons as compared with astrocytes (Fig. 2a left), whereas no differences were found at the Beta-actin gene promoter (Fig. 2a right). We next investigated whether Nrf2 promoter hypo-acetylation and transcriptional repression could be reversed by inhibiting histone deacetylases. Treatment of neurons with the ClassI/II histone deacetylase (HDAC) inhibitor trichostatin A (TSA) boosted Nrf2 promoter H3 acetylation (Fig. 2b left), without affecting H3 acetylation at the Beta-actin promoter (Fig. 2b right), and resulted in increased Nrf2 expression (Fig. 2c), an effect also observed in hESC-derived neurons (Fig. 2d). In contrast, TSA did not increase Nrf2 expression in astrocytes of mouse or hESC origin (Supplementary Fig. 2a,b). TSA administration *in vivo* to adult mice also boosted Nrf2 expression in NeuN^+^ (Fig. 2e) but not NeuN^−^ cortical cells (Supplementary Fig. 2c). Thus, neuronal Nrf2 is amenable to derepression by HDAC inhibition *in vitro, in vivo* and in human neurons. This raised the question as to whether neuronal responsiveness to Nrf2 activators could also be restored. Indeed, the Nrf2 target gene Srxn1 was induced by tBHQ treatment in neurons that had been pre-treated with TSA (Fig. 2f). Thus, epigenetic repression of the Nrf2 promoter is a major reason why there is an absence of a functional Keap1-Nrf2 pathway in neurons, and reactivation of the pathway by HDAC inhibition is achievable.

### Neuronal Nrf2 repression is developmentally regulated

**Figure 3:**
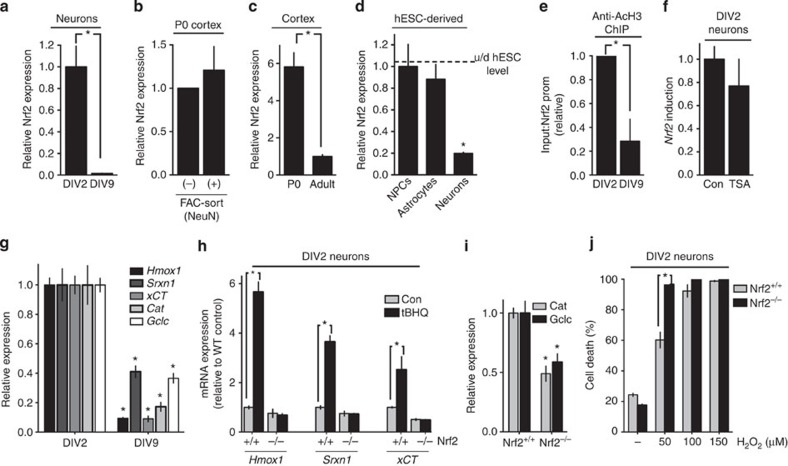
Nrf2 is epigenetically suppressed during early neuronal development. (**a**) Neuronal *Nrf2* expression measured at DIV2 and DIV9. *P<0.05, (*n*=4). (**b**) *Nrf2* mRNA expression analysis in NeuN^−^ versus NeuN^+^ cells obtained from acutely dissociated P0 mouse cortices by FACS (*n*=5). (**c**) Whole cortex *Nrf2* expression in P0 pups compared with adult mice. *P<0.05 (*n*=4 (adult), 6 (P0)). (**d**) A comparison of *NRF2* mRNA expression in neurons and astrocytes derived from hESCs, compared with neural precursor cells (NPCs) and undifferentiated (u/d) hESCs (dashed line). *P<0.05 (*n*=5 (NPCs), *n*=3 (neurons and astrocytes)). (**e**) Acetyl-H3 ChIP analysis of the Nrf2 promoter at DIV2, compared with DIV9. *P<0.05, (*n*=5). (**f**) TSA treatment of DIV2 neurons does not increase *Nrf2* mRNA expression (*n*=4). (**g**) Expression of Nrf2 target genes (analysed by qRT–PCR) compared between DIV2 and DIV9 neurons. *P<0.05 (*n*=4). (**h**) Analysis of the indicated Nrf2 target genes in Nrf2^+/+^ and Nrf2^−/−^ DIV2 neurons treated with vehicle or tBHQ suggests a functional Nrf2 pathway at this stage. *P<0.05 (*n*=5 (wild type (WT)), 4 (knockout (KO)). (**i**) Analysis of *Cat* and *Gclc* expression in Nrf2^+/+^ and Nrf2^−/−^ DIV2 neurons. *P<0.05 (*n*=5 (WT), 4 (KO)). (**j**) Quantification of H_2_O_2_-induced cell death in Nrf2^+/+^ and Nrf2^−/−^ DIV2 neurons. *P<0.05 (*n*=4, 1,700–4,500 cells analysed per condition per genotype).

As Nrf2 is widely considered to be ubiquitously expressed, its repression in neurons is unusual, raising the question as to whether the Nrf2 shut-off might be mapped to a specific developmental window. We observed that highly immature 2 days in vitro-DIV2 neurons expressed high levels of Nrf2 mRNA, compared with DIV9 (Fig. 3a). *In vivo*, P0 FAC-sorted NeuN^+^ and NeuN^−^ cells expressed similar amounts of Nrf2 mRNA (Fig. 3b). As Nrf2 mRNA levels overall (in total cortical homogenate) were found to greatly exceed those in the P0 cortex than in the adult (Fig. 3c), it is apparent that P0 cortical neurons express high levels of Nrf2 *in vivo*, as compared with adult neurons. Moreover, in our hESC system, we compared Nrf2 mRNA levels in hESC-derived cortical-patterned neurons with those in their direct precursors (hESC-derived neural precursor cells (NPCs)) as well as undifferentiated hESCs. We found Nrf2 mRNA levels are similar in undifferentiated hESCs, hESC-derived NPCs and, indeed, hESC-derived astrocytes (Fig. 3d). Only when NPCs are allowed to differentiate to neurons do levels of Nrf2 markedly decline (Fig. 3d). Thus, *in vitro, in vivo* and in a human stem cell-based system, neuronal Nrf2 expression becomes repressed during a developmental window early in neuronal commitment. The epigenetic repression of Nrf2 described in Fig. 2 takes place during the early stages of development. In agreement with this hypothesis, ChIP studies revealed a developmental drop in Nrf2 promoter histone H3 acetylation between DIV2 and DIV9 in neurons (Fig. 3e). Moreover, and in contrast to DIV9 neurons, TSA treatment of DIV2 neurons did not result in increased Nrf2 expression (Fig. 3f), indicative of a relatively active, unrepressed promoter at this developmental stage.

The developmental shut-off of Nrf2 expression is also associated with reduced expression of the Nrf2 target genes *Hmox1, Srxn1*, *xCt*, *Cat* and *Gclc* (Fig. 3g), strongly suggesting that very early in development, the Nrf2 pathway in neurons is actually functional. Indeed, we found this to be the case: induction of Nrf2 target genes in response to tBHQ treatment was observed in Nrf2^+/+^ DIV2 neurons but not in DIV2 Nrf2^–/–^ neurons (Fig. 3h). Moreover, further comparison of Nrf2^+/+^ and Nrf2^–/–^ neurons at DIV2 revealed that basal Nrf2 activity supported *Cat* and *Gclc* expression (Fig. 3i), and that Nrf2^–/–^ neurons showed a modest increase in vulnerability to oxidative insults (Fig. 3j). Thus, very young neurons possess a functional Nrf2 pathway that contributes to their intrinsic antioxidant defences, which becomes lost during maturation.

### Nrf2 antagonizes maturation of developing cortical neurons

**Figure 4:**
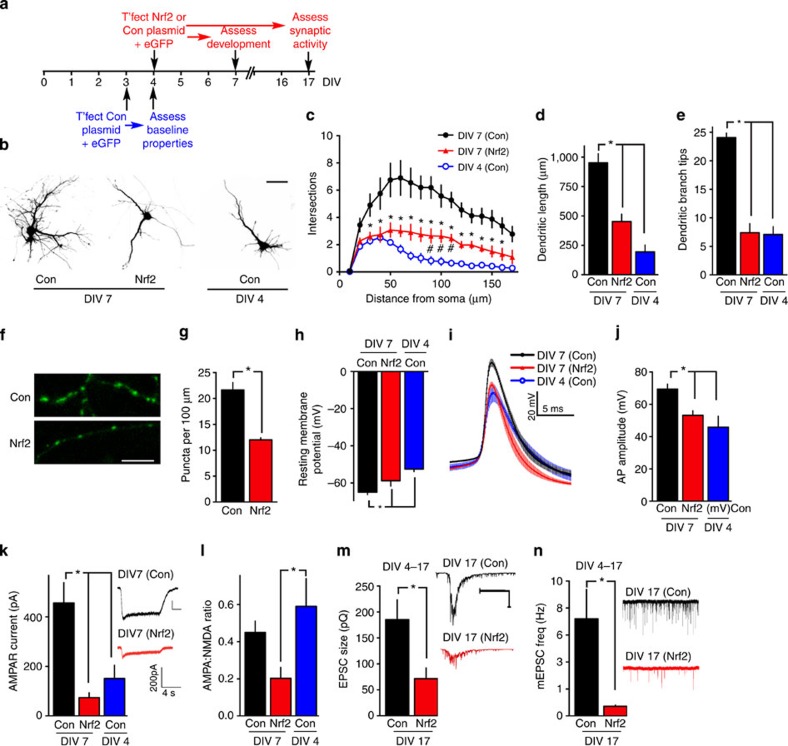
Forced Nrf2 expression in young neurons impairs development. (**a**) Schematic demonstrating the experimental timeline. Neurons were developmentally assessed (either morphologically or electrophysiologically) on DIV7 or DIV17 (for synaptic activity). For baseline DIV4 neuronal morphology and electrophysiological properties, neurons were transfected on DIV3 and assessed 24 h later. (**b**) Example images of transfected neurons on either DIV7 or DIV4 as indicated. Scale bar, 50 μm. (**c**) Sholl analysis of dendritic morphology of control (DIV4 and DIV7) and Nrf2-transfected (DIV7) neurons. Two-way analysis of variance plus Tukey’s *post-hoc* test here and throughout the figure. *P<0.05 compared with DIV7 (control (Con)); ^#^*P*<0.05 compared with DIV4 (Con); 40–48 neurons analysed per condition within *n*=4 independent experiments. (**d**,**e**) Dendritic length (**d**) and branch tip number (**e**) were analysed in the population of neurons studied in **c**. *P<0.05 (*n*=4). (**f**) Example images of PDS-95 puncta in DIV8 neurons transfected with YFP-tagged PSD-95 plus either a plasmid encoding Nrf2 (pNrf2) or control plasmid on DIV4 (scale bar, 10 μm). (**g**) Quantification of **f** showing a reduction in PSD-95 density in pNrf2-transfected DIV8 neurons (*P<0.05, dendrites from 24 to 30 neurons were studied for each condition within *n*=3 independent experiments). (**h**) Neuronal Nrf2 expression between DIV4–7 causes a significant change in resting membrane potential, as compared with controls (*P<0.05, *n*=11,9,7). (**i**) Example whole-cell current-clamp traces of evoked action potentials (APs) recorded from neurons transfected as indicated. (**j**) Quantification of AP amplitude in neurons transfected as indicated (*P<0.05, *n*=9,9,8). (**k**) AMPA receptor currents measured in DIV7 and DIV4 neurons transfected as indicated (*P<0.05, *n*=11,10,8). (**l**) The AMPA/NMDA ratio was calculated in neurons expressing pNrf2 as compared with control plasmid (*P*<0.05, *n*=11,10,8). (**m**) Neurons were transfected with either control plasmid or pNrf2, plus an eGFP co-transfection marker on DIV4. On DIV17, EPSCs were recorded over a 5-min period and the average charge transfer (pQ) calculated. *P<0.05 (*n*=8). Example traces are shown to the right. Scale bar, 500 pA, 500 ms. (**n**) mEPSC frequency measured in neurons transfected as indicated on DIV4 and recorded on DIV17. *P<0.05 (*n*=9 control, *n*=8 Nrf2).

To investigate the biological purpose served by the developmental silencing of neuronal Nrf2, we studied the impact of forced expression of Nrf2 during the developmental period in which it is ordinarily shut-off (Fig. 4a). Expression of Nrf2 at DIV4 resulted in impaired dendritic outgrowth and arborization, assayed at DIV7 by Sholl analysis, and by measuring total dendritic length and total dendritic branch tip number (Fig. 4b–e). A comparison of these properties with DIV4 neurons revealed that dendritic branching, which normally increases strongly during this developmental window (a threefold increase in branch number over this period), is halted by the forced expression of Nrf2 (Fig. 4e). We also studied postsynaptic density protein 95 (PSD-95) puncta, by expressing a YFP-PSD-95 fusion construct to label potential sites of nascent synaptogenesis, and found that forced Nrf2 expression diminished PSD-95 puncta density (Fig. 4f,g), further indicating an impairment of neuronal development and maturation. We also examined the influence of forced Nrf2 expression on the electrophysiological maturation of developing neurons between DIV4 and DIV7, a period where resting membrane potential hyperpolarizes (Fig. 4h) and action potential (AP) amplitude increases (Fig. 4i,j), together with an increase in the amplitude of α-amino-3-hydroxy-5-methyl-4-isoxazolepropionic acid receptor (AMPAR) mediated currents (Fig. 4k). Strikingly, we found that all of these developmental changes were strongly retarded by ectopic Nrf2 expression (Fig. 4h–k). Interestingly, not all developmental processes were equally affected: in particular, AMPAR-mediated currents were affected strongly (both net currents and current density (current/capacitance), Fig. 4k, Supplementary Fig. 3), and far more than *N*-methyl-D-aspartate receptor (NMDAR)-mediated currents. This resulted in a twofold reduction in AMPAR/NMDAR ratio, lower than that observed at either DIV7 or DIV4 (Fig. 4l). We next wanted to ascertain the impact of forced Nrf2 expression on synaptic currents. To study this we transfected neurons at DIV4 as before, but recorded from them at a later developmental stage (DIV17) to allow further synaptic maturation within the network. We first confirmed by immunofluorescence that Nrf2 was still expressed from the plasmid at DIV17 (data not shown) and that AMPAR current and current density were reduced (Supplementary Fig. 3b,c), as is the case at DIV7 (Fig. 4k and Supplementary Fig. 3a). To assess the magnitude of synaptic activity, we measured the average charge transfer during a spontaneous polysynaptic excitatory postsynaptic currents (EPSCs), and found that in Nrf2-expressing cells the EPSC size was far lower than in control-transfected neurons (Fig. 4m), consistent with there being fewer functional synapses. In addition, we analysed the influence of spontaneous tetrodotoxin-insensitive miniature EPCSs (mEPSCs). mEPSC frequency offers a functional measure of synapse number. We found that Nrf2 expression caused a strong reduction in mEPSC frequency (Fig. 4n) but not size (Supplementary Fig. 3j), consistent with there being fewer functional synapses, in agreement with the YFP-PSD-95 data (Fig. 4g) and the tetrodotoxin-sensitive synaptic EPSC results (Fig. 4m). Collectively, these data provide functional evidence that synaptic development is retarded by Nrf2.

Next we investigated whether forced expression of Nrf2 in more mature neurons that are developing less rapidly may be less deleterious to neuronal function. We investigated the influence of Nrf2 expression between DIV14 and 17, which provides a 3-day expression window similar to the DIV4–7 window used in the earlier assessments. In contrast to DIV4–7, when AMPAR currents are increasing dramatically (Fig. 4k), AMPAR currents have stabilized by DIV14 (Supplementary Fig. 3d). Interestingly, we found that Nrf2 expression during DIV14–17 did not influence AMPAR currents (Supplementary Fig. 3e, expression of Nrf2 was confirmed (data not shown)). Dendritic analysis is problematic at DIV17 due to extensive, overlapping processes from neighbouring transfected cells. However, measurement of membrane capacitance provides a surrogate measure of membrane area (and therefore outgrowth). Consistent with the dendrite analysis data (Fig. 4b–e), membrane capacitance increases sharply between DIV4 and 7, and this increase is inhibited by Nrf2 (Supplementary Fig. 3f). In contrast, membrane capacitance is stable between DIV14 and 17 (Supplementary Fig. 3g) and Nrf2 expression between DIV14 and 17 has no effect compared with control-transfected neurons (Supplementary Fig. 3h). Of note, Nrf2 expression over an extended window (DIV4–17) does reduce membrane capacitance (Supplementary Fig. 3i), just as it impairs synaptic maturation (Fig. 4m).

Collectively, these data indicate that forced expression of Nrf2 in neurons early in differentiation (around DIV4) at a time when it is normally subject to repression is deleterious to their development. These developmental defects become apparent quickly (by DIV7), potentially because growth and electrophysiological maturation is rapid at this stage. Moreover, these defects are still observed later on (DIV17) so long as Nrf2 expression is initiated early, but not if expression is initiated later in development (DIV14). We suggest that downregulation of Nrf2 early in development might help establish a redox environment, which is permissive for signalling pathways involved in neuronal maturation during a critical developmental window over which particularly rapid electrical and morphological maturation takes place.

### Developmentally important pathways are repressed by Nrf2

**Figure 5:**
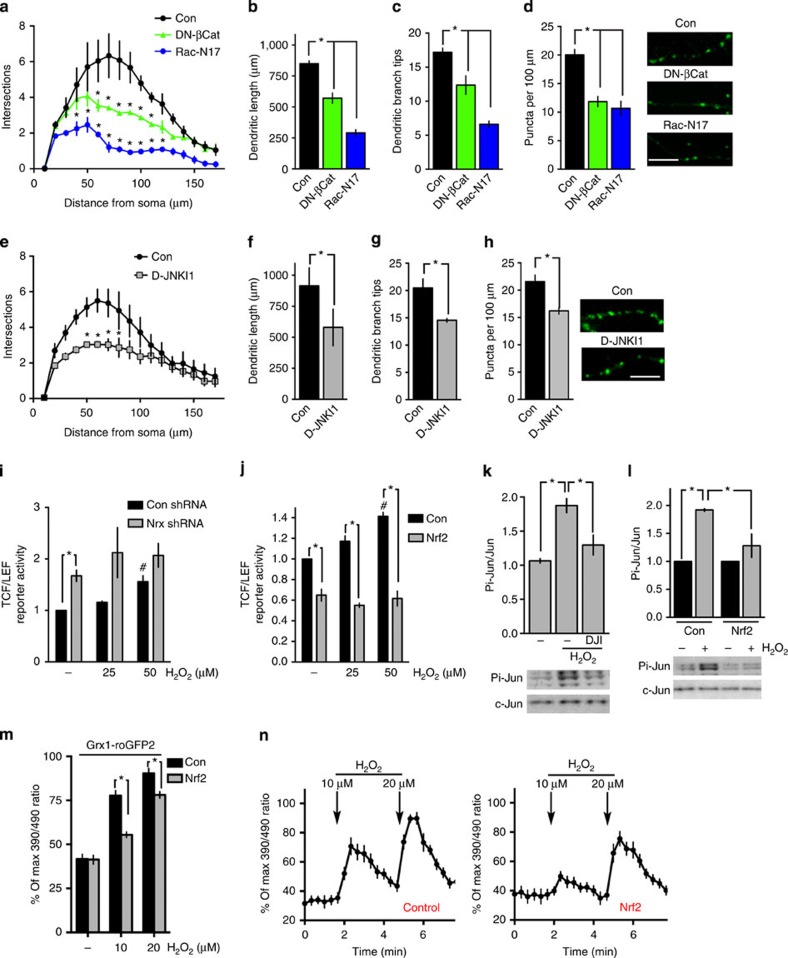
Nrf2 inhibits redox-sensitive neuronal developmental signalling pathways. (**a**) Sholl analysis of neurons transfected DIV4–7 with DN-βCat: βcat ARM-Myc; Rac-N17: Rac N17-Myc. *P<0.05 compared with control (Con): two-way analysis of variance (ANOVA) plus Dunnett’s *post-hoc*, *n*=3, 24 cells per condition. (**b**,**c**) Total dendritic length (**b**) and branch tip number (**c**) calculated for the cells analysed in **a**. *P<0.05, one-way ANOVA plus Dunnett’s *post-hoc*. (**d**) YFP-PSD-95 puncta density measured. *P<0.05, *n*=4 (32–40 cells quantified per condition). Example pictures to the right (scale bar, 10 μm). (**e**) Sholl analysis of neurons treated daily±D-JNKI1 (2 μM) DIV4–6. *P<0.05, two-way ANOVA plus Sidak’s *post-hoc*, *n*=4 (40 cells per treatment). (**f**,**g**) Dendritic length (**f**) and branch tip number (**g**) measured in the cells treated as in **e**. *P<0.05 Student’s *t*-test, *n*=4 (20 cells per treatment). (**h**) YFP-PSD-95 puncta density measured. *P<0.05 (*n*=3, 24 cells per condition). Example pictures to the right (scale bar, 10 μm). (**i**) Neurons were transfected with a TCF/LEF-luciferase reporter of Wnt signalling, a pTK-renilla Con, Dvl1 plasmid, plus Con or Nrx-directed shRNA vector (ratio: 2:1:2:2). 48 h post-transfection, neurons were treated with H_2_O_2_ for 16 h, and reporter activity measured (normalized to Renilla). *P<0.05, *n*=3: two-way ANOVA with Bonferroni’s *post-hoc* test here and for **j**. ^#^Difference between H_2_O_2_-stimulated values and the corresponding Con. (**j**) Neurons were transfected with the Wnt reporter, Renilla, Dvl1 plasmid, plus Con or Nrf2 vectors (ratio: 2:1:2:2). H_2_O_2_-induced Wnt activity measured as for **i**. *P<0.05, *n*=4. (**k**) Western analysis of H_2_O_2_ (25 μM)-induced c-Jun (Ser-73) phosphorylation±D-JNKI-1 (DJI) pretreatment. *P<0.05 (*n*=3). (**l**) Western analysis of H_2_O_2_-induced c-Jun (Ser-73) phosphorylation in neurons nucleofected with Con or Nrf2-encoding plasmids. *P<0.05, two-tailed *t*-test (*n*=3). (**m**,**n**) Nrf2 activity buffers neuronal redox potential. Neurons expressing Grx1-roGFP plus either Con (globin) or Nrf2-encoding plasmids were imaged during H_2_O_2_ treatment. Ratios (ex=387±5 and 494±10; em=530±10 in both cases) for each cell were calculated and normalized to the maximal ratio obtained by treating cells with high H_2_O_2_ (100 μM). *P<0.05, two-way ANOVA, plus Bonferronni’s *post-hoc*, *n*=6 (30 (Con) and 35 (Nrf2) cells analysed). (**n**) Example traces of single experiments relating to **m**.

The Wnt signalling pathway plays a key role in neuronal differentiation^23^; both canonical (via β-catenin) and non-canonical (via Rac and JNK) Wnt signalling contribute to dendritic development and synaptogenesis^24^,^25^. Consistent with this, we found dominant negative mutants of β-catenin (β-cat-ARM-Myc) or Rac (Rac-N17) interfered with dendritic development (Fig. 5a–c), with Rac-N17 having a considerably stronger effect (Fig. 5a–c). The highly specific peptide inhibitor D-JNKI-1 also impaired dendritic outgrowth and arborization (Fig. 5e–g). PSD-95 clustering was also impaired by interfering with β-catenin, Rac or JNK (Fig. 5d,h). In addition, expression of Rac-N17, or JNK inhibition, also resulted in lower AMPAR currents (Supplementary Fig. 4a). Thus, cortical neuronal development is impaired by inhibition of Rac and JNK, mediators of non-canonical Wnt signalling, as well as via the interference of canonical Wnt signalling via inhibition of β-catenin, consistent with previous findings in hippocampal neurons^23^,^24^,^25^.

The Wnt pathway is known to be sensitive to cellular redox status, and can be potentiated by ROS (H_2_O_2_) at least in part via inactivation of the Dishevelled (Dvl) inhibitor nucleoredoxin (Nrx), which is redox-sensitive^26^. However, Wnt pathway regulation by ROS in neurons is not well-studied. Moreover, a recent study demonstrated that ROS generation is important for Wnt-dependent differentiation in a neural progenitor cell line, as well as for the dissociation of Nrx from Dvl^27^. To investigate potential redox regulation of Wnt signalling in cortical neurons, we used a mixed culture preparation (90% neurons, 10% astrocytes) in which doses of up to 50 μM H_2_O_2_ were not neuro-toxic, and a transfection protocol that overwhelmingly targets neurons over astrocytes^28^. We found that mild sub-toxic doses of H_2_O_2_ potentiated Wnt signalling as assayed by measuring β-catenin-dependent TCF/LEF-luciferase reporter activity (M50 Super 8x TOPFlash β-catenin reporter, Fig. 5i,j). It seems likely that Nrx is involved in this potentiation because Nrx-directed short hairpin RNA (shRNA) both increased basal Wnt pathway activity and partly occluded the potentiating effect of mild sub-toxic doses of H_2_O_2_ (Fig. 5i). Forced expression of Nrf2 both lowered basal Wnt reporter activity, and abolished its potentiation by H_2_O_2_ treatment (Fig. 5j). We also observed that the same low H_2_O_2_ conditions that boosted Wnt signalling also served to activate JNK signalling, again in a manner inhibited by Nrf2 expression (Fig. 5k,l). It should be recognized, however, that JNK also has other redox-sensitive upstream activators such as ASK1. Nevertheless, these data show that developmentally important signalling pathways are sensitive to the redox status of maturing neurons, and are inhibited by ectopic Nrf2. This suggests that Nrf2 expression boosts antioxidant levels to such a point that low ROS levels are unable to activate redox-sensitive signalling pathways because they are rapidly inactivated in the cell. To visualize this directly, we employed the Grx1-roGFP genetically encoded reporter of cellular redox potential, Grx1-roGFP2^29^, which is highly sensitive to small fluctuations in redox potential^29^. Small doses of H_2_O_2_ produced strong fluctuations in the probe signal (Fig. 5m,n, Supplementary Fig. 4c). In Nrf2-expressing neurons, the same doses of H_2_O_2_ elicited far weaker responses, conclusively showing that Nrf2 expression boosts the capacity of neurons to buffer their redox potential, providing an explanation for Nrf2’s inhibitory effects on H_2_O_2_-induced activation of JNK and Wnt signalling.

**Figure 6:**
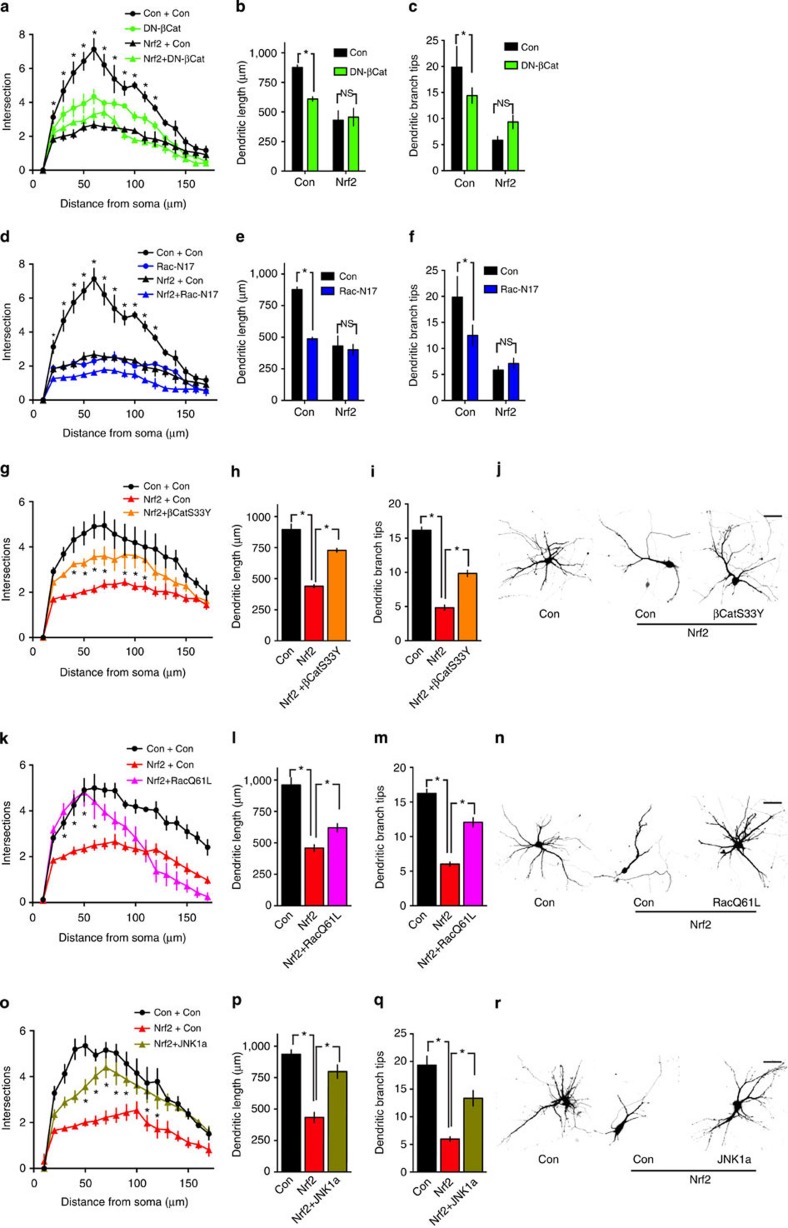
Activation of the JNK or Wnt pathways rescues detrimental Nrf2-dependent alterations in neuronal maturation. (**a**–**f**) Expression of Nrf2 occludes the deleterious effects of DN-βCat or Rac-N17 on neuronal development. Neurons were transfected on DIV4 with the indicated plasmids, plus GFP to visualize the cells, and studied at DIV7. Where used, quantities of DN-βCat, Rac-N17 and Nrf2 were always kept constant, and control plasmid used to ensure that total quantity of transfected DNA was also constant. (**a**,**d**) Sholl analysis of neurons transfected as indicated. *P<0.05, two-way analysis of variance (ANOVA), plus Tukey’s *post-hoc* test: *indicates difference between equivalent ‘Con’ and ‘Nrf2’ conditions. 15–24 cells analysed within *n*=3 independent experiments. Note that the same ‘Con’ and ‘Nrf2’ conditions are shown for **a** and **d** because the DN-βCat and Rac-N17 experiments were performed at the same time, with the data separated for easier visualization. (**b**,**c**,**e**,**f**) Dendrite length (**b**,**e**) and branch tip number (**c**,**f**) were calculated in neurons transfected with the indicated plasmids. *P<0.05, two-way ANOVA with Tukey’s *post-hoc* test; 15–24 cells analysed per condition within *n*=3 experiments. (**g**–**r**) Activation of Wnt, JNK or Rac signalling rescues the detrimental impact of Nrf2 expression on neuronal morphology. DIV4 neurons were transfected with the indicated plasmids and Sholl, dendritic length and branch tip analysis performed as previously. (**g**,**k**,**o**) Sholl analysis: *P<0.05, two-way ANOVA with Bonferronni’s *post-hoc* test. *A significant rescue, comparing the Nrf2 only condition to Nrf2+βCatS33Y or RacQ61L, or JNK1a (*n*=4, 32–36 cells analysed in total per condition). (**h**,**i**,**l**,**m**,**p**,**q**) *P<0.05, one-way ANOVA with Dunnett’s *post-hoc* test (*n*=4, 32–36 cells analysed in total per condition). (**j**,**n**,**r**) Example images of neurons analysed. Scale bar, 50 μm.

Collectively, these observations raise the possibility that the effects of Nrf2 expression during development are partly due to interference with Wnt-dependent development, the importance of which we have confirmed above. To investigate this we tested whether either dominant-negative β-cat-ARM or Rac-N17 expression had any influence on neuronal development against a background of forced Nrf2 expression, focusing on dendritic outgrowth and arborization. We found that the effect of either dominant-negative β-cat-ARM or Rac-N17 on dendritic arborization were entirely non-additive to the effects of Nrf2 expression (Fig. 6a–c and Fig. 6d–f respectively). This is suggestive of a common pathway: that is, that the effects of forced Nrf2 expression are due in part to Wnt pathway inhibition. Furthermore, these data also suggest that a key reason for Nrf2 shut-off during development is to allow Wnt-dependent development and maturation processes to take place.

### Rescue of developmental deficits induced by Nrf2

Finally, we investigated whether the deleterious effects of Nrf2 expression on dendritic outgrowth and arborization could be rescued by artificially activating canonical and non-canonical Wnt signalling or JNK. We found that expression of both a constitutively active form of β-catenin (β-catenin-S33Y, Fig. 6g–j) or Rac (Rac1-Q61L, Fig. 6k–n) or overexpression of JNK1 (Jnk/1a, Fig. 6o–r) partly rescued neurons from the morphological deficits caused by Nrf2 expression. Nrf2 expression was not affected (Supplementary Fig. 4e). Nrx shRNA also partly rescued dendritic length, but not branch tip number (Supplementary Fig. 4f). Collectively, these data support a model whereby Nrf2-driven antioxidant defences repress the activity of redox-sensitive Wnt and JNK pathways, leading to deficits in neuronal development. They also offer a biological reason behind the developmental epigenetic shut-off of the Nrf2 promoter: by slightly weakening neurons’ intrinsic antioxidant defences via Nrf2 inactivation, a more permissive environment is provided for redox-sensitive signalling pathways critical for neuronal development.

## Discussion

We have shown that Nrf2 expression is shut-off during neuronal development, contributing to weakened antioxidant defences, and have provided an explanation for the paradoxical inactivation of this cytoprotective pathway by demonstrating the inhibitory effect of Nrf2 on key developmental processes and pathways.

Cellular antioxidant defences must be carefully tuned to the requirements of the cell. Not only does the redox potential of the cell need to be appropriate for the stability of proteins and other molecules, but also the redox buffering capacity of the cell must strike a balance between limiting excessive ROS levels, while allowing redox signalling for physiological processes^30^. In the latter case, there is evidence that redox signalling directs differentiation and development in the nervous system and elsewhere. Mitochondrially derived ROS are important for adipocyte differentiation^31^ and are involved in promoting the oxidation of Nrx and activation of Wnt-dependent epidermal differentiation and hair follicle development^32^. Interestingly, ectopic expression of the *Drosophila* Nrf2 homologue CncC in the compound eye causes a rough-eye phenotype^33^, consistent with excessive Nrf2-induced activity being deleterious for normal differentiation and development.

There is growing evidence supporting a role for ROS in promoting neurogenesis and neuronal differentiation^34^,^35^,^36^. Differentiation of PC12 cells, Neuro2a cells, the human neural progenitor ReNcell VM197 line and also of rat cortical precursors is associated with ROS generation and is affected by antioxidant treatment^27^,^34^,^36^. Mechanistically, a link between ROS signalling and JNK-dependent synapse development has been demonstrated in the context of *Drosophila* neuromuscular junction development^37^. Moreover, it is apparent that physiologically appropriate levels of ROS must be maintained, as excessive oxidative stress can cause synapse overgrowth^37^. In addition to synaptic growth, JNK plays roles in dendritic arborization and axon formation, in part by controlling microtubule dynamics^38^. As a downstream effector of non-canonical Wnt signalling as well as redox-sensitive upstream activators such as ASK1, JNK is potentially regulatable by fluctuations in cellular redox potential via a variety of mechanisms. Wnt signalling is another developmental pathway potentiated by mild ROS exposure^26^,^27^. In neurons, Wnt signalling directs progenitor differentiation, dendritic development and synaptogenesis^23^,^24^,^25^,^27^ via both canonical and non-canonical pathways. Moreover, Rac, part of the non-canonical pathway, promotes AMPAR clustering and excitatory neurotransmission^39^. Our study indicates that these and potentially other pathways are inhibited by Nrf2-mediated gene expression in developing neurons, providing a biological reason behind the developmental shut-off of Nrf2 expression. Our working hypothesis is that Nrf2-directed antioxidant gene expression limits redox fluctuations that help to potentiate redox-sensitive signalling pathways. Two recent studies have shed light on the possible sources of these redox fluctuations. Bursts of mitochondrial superoxide production, mediated by transient mitochondrial permeability transition pore opening and caused by Ca^2+^ signals, have been implicated in promoting ROS-dependent neural progenitor differentiation^36^. The key trigger for this may be mitochondrial Ca^2+^ uptake, which has been shown to be critical for mitochondrial ROS production and ROS-dependent neural progenitor differentiation in a separate study^27^. However, beyond redox control of key redox-sensitive signalling pathways, it remains possible that Nrf2 may control the expression of genes that directly interfere with neuronal differentiation.

Activation of Nrf2-mediated transcription in astrocytes, either via forced Nrf2 expression or treatment with small-molecule Keap1 inhibitors, confers protection on nearby neurons^4^,^7^,^40^. This involves the production and release of astrocytic glutathione^4^, a process that is conserved in human stem cell-derived astrocytes^41^. Astrocytic Nrf2 is able to ameliorate pathology in models of a variety of neurodegenerative diseases, including ALS and Parkinson’s disease^7^,^16^,^40^,^42^. It had been reported, using an ARE-reporter gene that neurons respond less well to Nrf2 activators than astrocytes, the basis for which was unclear^4^. This finding is consistent with our observations that neither pharmacological inducers of Nrf2 nor genetic ablation of Keap1 can induce Nrf2-mediated responses in cortical neurons, because of the transcriptional repression of the Nrf2 gene itself. Our work also demonstrates that Nrf2 repression is also a feature of neurons *in vivo* (using acute FAC-sorting) and in neurons derived from hESCs. Mechanistically, we showed that histone H3 associated with the *Nrf2* gene promoter becomes hypo-acetylated during development, resulting in transcriptional repression. However, Class I/II HDAC inhibition resulted in only partial derepression of *Nrf2*, suggestive of additional repression mechanisms. Interestingly, the polycomb group protein EZH2, a histone H3 Lys27 (H3K27) trimethyltransferase, was recently identified as a negative regulator of Nrf2 expression in lung epithelial cells, raising the possibility that this may contribute to Nrf2 repression in neurons. The potential role of Class III HDACs remains to be explored.

Activation of Nrf2 by mild oxidative stress is a classical cytoprotective response that enables antioxidant defences to be boosted under conditions of demand^11^. The shut-off of Nrf2 in maturing neurons means that this route is either unavailable to neurons, or of limited relevance. However, other adaptive strategies exist to help match antioxidant capacity to oxidant exposure. Mild oxidative stress, and sublethal episodes of ischaemia *in vitro* and *in vivo* can activate Nrf2-dependent gene expression in astrocytes, which contribute to neuroprotective preconditioning *in vitro*^43^,^44^. Synaptic activity, an energetically expensive process that places metabolic demands on the cell and increases ROS production^45^ also triggers the expression of antioxidant genes, boosting intrinsic antioxidant defences^28^,^46^, including that of the glutathione (GSH) system^47^. Of note, some of these genes are known Nrf2 target genes but are induced in neurons via Nrf2-independent mechanisms involving factors AP-1 and ATF4 (refs 18, 28, 48). Moreover, neuronal activity has recently been reported to increase Nrf2 protein accumulation in astrocytes^49^. In addition, other neuroprotective transcription factors may help to compensate for Nrf2’s repression, including nuclear factor-κB, whose activity increases during neuronal maturation and which controls anti-apoptotic gene expression as well as MnSOD^50^,^51^,^52^. These and other mechanisms may have evolved to help mitigate the effects of low Nrf2 expression in neurons.

Another important question is whether (re)activation of the Nrf2 pathway in mature neurons offers a viable neuroprotective strategy. In the context of antioxidant defences, Nrf2 activation in neurons is likely to be beneficial, rendering neurons resistant to oxidative insults as well as ischaemic-like conditions *in vitro*. Moreover, we observed that forced Nrf2 expression retarded neuronal development DIV4–7 but not when initiated over a later time window (DIV14–17). Nevertheless, further investigations are required (and planned) to determine whether prolonged Nrf2 activation in mature neurons would have deleterious functional consequences. In addition to being involved in synaptogenesis, the Wnt pathway has recently been implicated in synapse maintenance^53^, raising the possibility that perturbations that interfere with this pathway may functionally compromise mature neurons. If this were the case, then the continued repression of Nrf2 in mature neurons could be desirable, so that transcriptional responses in the brain to Nrf2-activating drugs are preferentially induced in non-neuronal cells. Alternatively, it could be that Nrf2 repression is needed only very early in development during a period of particularly rapid electrical and morphological maturation. One can speculate that during this early period, redox signalling is important for potentiating and maximizing the strength of developmental signalling pathways. However, in mature neurons there may not be a need for such a potentiation, and lower levels of pathway activity (for example, to sustain synapse maintenance) may suffice. If this is the case, then derepression of the neuronal Nrf2 promoter could be a promising therapeutic strategy for brain disorders associated with oxidative stress.

The data presented here suggests that derepression of the Nrf2 promoter by HDAC inhibition may render the neuronal Nrf2 pathway partly activatable by classical inducers, which block the actions of Keap1. Interestingly, while classical inducers such as tBHQ fail to induce a significant response in neurons, the enone-type electrophilic compound curcumin and its corresponding dienone, NEPP11 have been reported to activate Nrf2 target gene expression directly in neurons^54^,^55^. Of note, curcumin is known to inhibit Class I HDACs and DNA methylation (as well as being an activator of Nrf2 via blocking its Keap1-mediated degradation), raising the possibility that its action involves both derepression of the Nrf2 promoter and inhibition of Keap1-mediated protein degradation. Regardless, to fully derepress Nrf2, further research is necessary to uncover the molecular mechanism of its developmental repression. Neuron-specific chromatin remodelling and other epigenetic changes are known to be important for development^56^,^57^, so it is possible that Nrf2 is a target for these pathways. For example, Brg1/hBrm-Associated Factor (BAF) chromatin remodelling complexes are capable of both gene activation and repression, depending on their composition, and neuron-specific BAF complexes are critical for dendritic outgrowth and synaptogenesis^57^.

Of course, even if Nrf2 expression in neurons is derepressed, it still needs to be activated by an appropriate small molecule to mediate a response. Although several Nrf2-activating molecules have demonstrated efficacy in a variety of preclinical animal models of brain disease, BG-12 (dimethyl fumarate) has emerged as a particularly promising agent. BG-12 is a cytoprotective compound licensed for relapsing remitting multiple sclerosis, which activates Nrf2-mediated gene expression and has a mechanism of action (at least in animal models) that is dependent on Nrf2 (refs 58, 59). Alternatively, it could be that in targeting Nrf2 in neurons a balance might be struck between boosting the Nrf2 pathway while preventing an overloading of excessive redox-buffering enzymes, or in only targeting those cells that are stressed and in need of antioxidant support. To this end, the development of so-called ‘pathologically activated therapeutics’ in which oxidative stress modifies a pro-drug to become a functional Nrf2 activator may offer a promising strategy to activate Nrf2 only where it is needed^60^.

To conclude, the striking shut-down of the powerful Nrf2 cytoprotective pathway in neurons explains both the relative vulnerability of neurons to oxidative stress and their reliance on astrocytic support, but appears to be necessary for proper neuronal development.

## Methods

### Cell culture

Cortical cells were cultured from E17.5 CD1 mouse embryos, Nrf2^–/–^ and wild-type embryos (c57Blk/6), and Keap-1^–/–^ and wild-type embryos, essentially as previously described^61^,^62^. Briefly, cortices were dissociated in papain for 2 × 20 min and plated at a density of between 9 and 13 × 10^4^ neurons per cm^2^. Three cell culture types were prepared: mixed^28^ neuronal/astrocyte cultures (90% NeuN^+^ neurons and 10% GFAP^+^ astrocytes), highly enriched neuronal^43^ cultures (>98% NeuN^+^ neurons and <0.2% GFAP^+^ astrocytes) and highly enriched astrocyte^28^ cultures (>96% GFAP^+^ astrocytes). Mixed neuronal/astrocyte cultures and highly enriched neuronal cultures were prepared in Neurobasal growth medium plus 1% rat serum (Harlan Laboratories), B27 (Life Technologies Ltd), 1 mM glutamine and 1 × antibiotic/antimycotic (Life Technologies Ltd), whereas astrocyte cultures were obtained by plating cells at low density in DMEM+10% Fetal Bovine Serum and 1 × antibiotic/antimycotic (all Life Technologies Ltd). To prevent astrocyte proliferation in neuron-containing cultures, the anti-mitotic drug Cytosine β-D-arabino- furanoside hydrochloride (1.2 mM) was applied either immediately post plating (pure neuronal cultures) or on DIV4 (mixed cultures). Cultures were utilized as indicated between DIV3 and 17, and were fed with the above described appropriate growth medium on DIV4. Before experimentation cells were removed from growth medium and washed and placed in a minimal defined medium^63^ containing 10% minimum essential media (Life Technologies Ltd) and 90% Salt-Glucose-Glycine (SGG) medium, which is comprised of 114 mM NaCl, 0.219% NaHCO_3_, 5.292 mM KCl, 1 mM MgCl_2_, 2 mM CaCl_2_, 10 mM HEPES, 1 mM Glycine, 30 mM Glucose, 0.5 mM sodium pyruvate, 0.1% Phenol Red; osmolarity 325 mosm l^−1^) for at least 3 h. When cultures were used >DIV14, a 1:1 replacement of conditioned media with fresh growth media, which lacked serum but was supplemented with glucose (10 mM), was conducted on DIV9 and 12. On DIV14, SGG supplemented with Insulin-Transferrin-Selenium was used as the replacement media.

Establishment and maintenance of the hESC lines H9 and HUES9 (Harvard University, Cambridge, MA, USA) are as previously described^41^. Briefly, hESC lines were cultured and regularly passaged on a feeder layer consisting of irradiated mouse embryonic fibroblasts. The neuralization and differentiation of NPCs into neurons and astrocytes followed our established protocols^21^,^22^,^41^. Briefly, human PSCs were neurally converted (to NPCs) in suspension in chemically defined medium as described^21^,^22^. The media were changed to Base media (A-DMEM/F12, 1% P/S, 1% Glutamax, 1% N2), 0.4% B27, 2.5 ng ml^−1^ FGF2 upon observation of radially organized structures in neurospheres (10–21 days) and plated on Laminin (Sigma)-coated tissue culture plates (Nunc) a week later. Neural rosettes were mechanically isolated, dissociated with Accutase (Sigma) and 20–40 k cells were plated in one Laminin-coated well of a 96-well plate in proliferation media (Base media, 0.1% B27, 10 ng ml^−1^ FGF2 and 10 ng ml^−1^ EGF where stated). aNPCs were grown to high density before passaging 1:2 with Accutase on laminin-coated plates until passage 5–6 and maintained on 1:100 Reduced-growth factor Matrigel (BD Biosciences)-coated plates thereafter. For neuronal differentiation, NPCs were plated in default media (A-DMEM/F12, 1% P/S, 0.5% Glutamax, 0.5% N2, 0.2% B27, 2 μg ml^−1^ Heparin (Sigma)) on poly-D-lysine (Sigma), laminin (Sigma), fibronectin (Sigma) and Matrigel-coated coverslips for differentiation and fed twice a week. Default media were supplemented with 10 μM forskolin (Tocris) in weeks 2 and 3. From week 4 onwards, forskolin was removed and default media were supplemented with 5 ng ml^−1^ brain-derived neurotrophic factor and 5 ng ml^−1^ glial-derived neurotrophic factor. For astrocytic differentiation, NPCs were plated in modified basal plating medium supplemented with BMP2 and BMP4 (R&D) at 10 ng ml^−1^ and Leukemia Inhibitory Factor (Sigma) at 20 ng ml^−1^.

### Transfection and plasmids

Neuronal transfections were carried out using Lipofectamine 2000 (2.33 μl per well, 1 μg ml^−1^, Life Technologies Ltd) on DIV 3, 4 and 8 neurons. For neurons subject to oxygen glucose deprivation (OGD), transfections were carried out in a trophically deprived transfection medium (TMo, 90% SGG+10% minimum essential media^63^). Non-OGD-related transfections were carried out in normal transfection medium (TMits, TMo plus an Insulin-Transferrin-Sodium Selenite Media Supplement). As reported previously, the transfection efficiency for this protocol is approximately 5% for mixed cultures, with 99% of the total transfected cells being neuronal in nature, as determined by NeuN staining^19^. Constructs were co-transfected with peGFP in order to identify successfully transfected cells. To ensure that GFP-positive neurons also express the plasmid of interest, a favourable ratio was used (enhanced GFP-encoding plasmid (pEGFP)/plasmid of interest, 1:2), as previously validated in the case of RFP-encoding plasmid (pRFP)^28^. In all experimental comparisons, the amount of DNA transfected per plasmid of interest as well as the total amount of DNA from all plasmids (0.6–0.7 μg cDNA per well) was identical, with control plasmid being utilized to balance amounts where required. Transfections were carried out using Lipofectamine as previously described^63^, with cells incubated with transfection mixture for 3 h before removal and transfer back to pre-transfection incubation medium. Transfections designed for morphological assessment of neurite outgrowth were performed at a lower dilution (1/16 of the normal full strength, diluted in TMits) in order to reduce the total number of transfected cells. This enabled accurate tracking of neurite projections from a single cell despite the high density of the culture. The diluted DNA/lipofectamine transfection mixture was left on for 3 h as with other full-strength transfections. For transfections on younger neurons (DIV3–4), cells were returned to growth medium, and fed on DIV 4 following the termination of the transfection protocol as usual. Neurons were then assessed on the indicated day as appropriate. In all instances the utilized control plasmid was β-globin, referred to as control plasmid (here and throughout). Plasmids expression was confirmed (Supplementary Fig. 4d) and have been previously described: pEF-Nrf2 (ref. 64), M50 Super 8x TOPFlash^65^, pcDNA3-S33Y β-catenin-Flag^66^, pcDNA3-EGFP-Rac1-Q61L-myc, β-catenin-ARM-myc^67^, Rac N17-myc^68^, pCDNA3-Jnk1a1 (ref. 69), NRX-RNAi in pSuper and control-RNAi in pSuper^26^, Grx1-roGFP^29^, DVl1-HA^25^. The PSD-95-YFP vector was kindly provided by Noboru Komiyama.

### Electrophysiological recording and analysis

Coverslips containing cortical DIV4–17 neurons were transferred to a recording chamber perfused (at a flow rate of 3–5 ml min^−1^) with an external recording solution composed of (in mM): 150 NaCl, 2.8 KCl, 10 HEPES, 2 CaCl_2_, 1 MgCl_2_, 10 glucose, 0.1 glycine and 0.002 strychnine, pH 7.3 (320–330 mOsm). Patch-pipettes were made from thick-walled borosilicate glass (Harvard Apparatus, Kent, UK) and filled with a K-gluconate-based internal solution containing (in mM): 155 K-gluconate, 2 MgCl_2_, 10 Na-HEPES, 10 Na-PiCreatine, 2 Mg_2_-ATP and 0.3 Na_3_-GTP, pH 7.3 (300 mOsm). To ensure sufficient voltage-clamp of EPSCs, QX-314 (5 mM) was included in the internal solution to block voltage-gated conductances. Electrode tips were fire-polished for a final resistance ranging between 5 and 10 MΩ. Currents were recorded at room temperature (21±2 °C) using either an Axopatch-1C or Axon Multiclamp 700B amplifier (Molecular Devices). Neurons were voltage-clamped at –60 mV, and recordings were rejected if the holding current was greater than –100 pA or if the series resistance drifted by more than 20% of its initial value (<30 MΩ). Whole-cell currents were analysed using WinEDR v3.2 software (John Dempster, University of Strathclyde, UK). To measure AMPA receptor-mediated currents, 50 μM S-AMPA (Tocris) was applied for 5–10 s to reach a steady-state plateau, washed out for 1–2 min, then re-applied to elicit a second response. The same procedure was used to acquire whole-cell NMDAR currents (100 μM NMDA, Tocris Bioscience), except using Mg^2+^-free external recording solution in which MgCl_2_ was substituted with 2 mM NaCl. All currents were quantified as a 1 s average of the steady-state plateau minus the baseline at the second agonist application, and normalized to the cell capacitance. Analysis of EPSC areas was carried out in OriginLab. mEPSC frequencies and amplitudes were analysed using MiniAnalysis (Synaptosoft) with the detection threshold set at –5 pA. To be included for analysis, mEPSCs needed to possess a monotonic rise and an exponential decay. Overall mEPSCs recorded from globin or Nrf2-transfected cells had mean 10–90% rise times of <1.5 ms and mean decay time constants of <3 ms.

### OGD insult and cell fate tracking

DIV 7 neurons were transfected with either pEF-Nrf2 or control plasmid, plus peGFP. On DIV9, GFP-expressing neurons were imaged using a Leica AF6000 LX imaging system and DFC350 FX digital camera, and their locations mapped using the Leica ‘Mark and Find’ software application. Once GFP^+^ neurons were identified, imaged and plate/well locations saved, they were returned to the incubator to re-equilibrate. Following a 3-h period of equilibration, neurons were then exposed to 3 h OGD. Cells were washed and incubated in a glucose-free, balanced salt solution (SGG with mannitol substituted for glucose), previously degassed with 95% N_2_–5% C0_2_ and placed in an anoxic modular incubator chamber for 3 h (as compared with cells washed and incubated in normoxic glucose-containing media). After 18–20 h (DIV 10), saved plate and well locations were reloaded and images of the same cells were captured. Cell death was assessed by counting the number of surviving GFP^+^ neurons pre- and post- exposure to OGD. The user was blind during both image capture and cell death analysis. In the vast majority of cases, neuronal death was easily identifiable by the replacement of a healthy GFP-expressing cell with the presence of fragmented neurites and fluorescent cell debris. Cell death is presented as the percentage of dead neurons/total post-OGD. Approximately 125 neurons were monitored for each plasmid across four independent experiments.

### Peroxide-induced cell death

Cultures were transferred to TMo for at least 3 h before the start of the experiment (DIV as indicated). Cells were then treated with H_2_O_2_ (dose as indicated) and cell death determined 24 h later via fixation with 4% formaldehyde and nuclear staining with 4-,6-diamidino-2-phenylindole (DAPI). As previously described^28^, images were captured and cell death quantified using an automated technique created in Image J, which takes advantage of the large difference in the nuclear size of living or dead cells. The automated system then yields the number of dead versus living cells, enabling the user to calculate the number of dead cells as a percentage of the total cell population. For DAPI-assisted automated quantification, cells were imaged under the × 10 objective, allowing ∼1,000 cells to be imaged at a time, with at least 12 images per treatment, allowing approximately 12,000 cells to be quantified per experiment, in at least three independent experiments. During both image capture and image quantification, the user was blind.

### Morphological assessment of neurite branching

Neurons were transfected with efGFP plus plasmid(s) of interest on DIV3/4 and left until DIV 4/7, whereupon they were fixed, stained (as described in immunofluorescence below) and then imaged at × 20 for morphological analysis. Images were subjected to Sholl analysis, as well as measurement of dendritic length and branch tip number, as facilitated by the use of Image J software. Sholl analysis provides an assessment of dendritic arborization and branch complexity via quantification of the number of intersecting branch points across concentric circles spaced at specific distances from the soma. Sholl analysis parameters were as follows: origin of the concentric radii was set at the midpoint of the longest axis of the soma, starting radius: 10 μm, ending radius: 170 μm, radius step size: 10 μm. Dendritic length represents the total summed length of all dendrite branches present. Branch tip number refers to the sum total of all terminal branch tips in a given neuron. In general, an average of eight to ten neurons were quantified for Scholl analysis, dendritic length and branch tip number for each investigated condition in a given experiment. Counts for the eight to ten cells were then averaged per biological replicate, with at least three replicates quantified in total.

### PSD-95 density analysis and quantification

DIV 4 neurons were transfected with the plasmid of interest plus YFP-tagged PSD-95, as described above. On DIV8, neurons were washed twice with pre-warmed TMO (to eliminate issues of autofluorescence associated with Neurobasal-A medium containing growth medium) and images of live PSD-95 expressing neurons were captured at × 20. PSD-95 density analysis was then performed on captured images using ImageJ software (Wayne Rasband, US National Institutes of Health, Bethesda, MD, USA). The use of immunostaining with antibodies against PSD-95 was deemed undesirable as staining intensity was too high to accurately separate distinct PSD-95 puncta. Live imaging of endogenous YFP signals from transfected YFP-tagged PSD-95 was optimal. Because of the impact on Nrf2 expression on dendritic branching, quantification of secondary dendritic branches was not always possible. In an effort to maintain consistency, the number of PSD-95 puncta along the full length of one dendritic branch (including primary and secondary sections) was totalled and then divided by the total length of the branch of interest. This density is presented as the total number of PSD-95 puncta per 100 μm, for simplicity. At least eight dendritic sections from independent cells were quantified per condition, with at least three biological replicates throughout all experiments.

### RNA isolation, reverse transcription (RT)–PCR and qPCR

RNA was isolated using the Roche High Pure RNA Isolation Kit (including optional DNase treatment), according to the manufacturer’s instructions (Roche). For quantitative PCR (qPCR), cDNA was synthesized from 1 to 3 μg RNA using the Roche Transcriptor First Strand cDNA Synthesis Kit, according to the manufacturer’s instructions. cDNA was then stored at −20 °C or immediately diluted (equivalent of 6 ng of initial RNA per 15 μl qPCR reaction, per gene of interest) for real-time PCR in a Stratagene Mx3000P QPCR System (Agilent Technologies), using the Roche FS universal SYBR Green MasterRox mix, according to the manufacturer’s instructions. The required amount of template was mixed with water, SYBR Green MasterRox mix and forward and reverse primers (200 nM each final concentration) to the required reaction volume. Technical replicates as well as no template and no RT negative controls were included and at least three biological replicates were studied per study. Primer sequences are described in Supplementary Table 1. The qRT-PCR cycling programme was 10 min at 95 °C; 40 cycles of 30 s at 95 °C, 40 s at 60 °C with detection of fluorescence and 30 s at 72 °C; 1 cycle (for dissociation curve) of 1 min at 95 °C and 30 s at 55 °C with a ramp up to 30 s at 95 °C (ramp rate: 0.2 °C s^−1^) with continuous detection of fluorescence on the 55–95 °C ramp. Data were analysed using the MxPro qPCR analysis software (Stratagene), with 18 s or GAPDH expression utilized as an internal normalizer. For murine experiments involving TSA application, expression of the housekeeping gene RPL13A, which encodes the ribosomal highly basic 23-kDa protein, was used as the internal standard for normalization, as RPL13A expression is unaffected by TSA application^70^.

### Chromatin immunoprecipitation

ChIP was performed using the MAGnify Chromatin Immunoprecipitation System (Life Technologies Ltd), according to the manufacturer’s instructions. Briefly 2 × 60 mm dishes were used per treatment (∼4.5 × 10^6^ cells). Cells were washed once with pre-warmed media and then incubated in media plus paraformaldehyde (1% final concentration) at room temperature for 8 min to enable protein/DNA cross-linking. Fixation was then quenched by addition of glycine (final concentration 125 mM, 5 min). Dishes were then transferred to ice, and cells washed and resuspended in ice-cold DNAse*-*free PBS, using plastic scrapers. Cell lysates were then transferred to microcentrifuge tubes and spun at 200*g* for 10 min to pellet. Following removal and discard of the supernatant, the cell pellet was resuspended (via mild pulsing on the vortex) in lysis buffer plus protease inhibitors, and left on ice for 5 min before being sonicated using a Branson 450 Sonifier (five 20 s pulses at 10% power, 15 s intervals between pulses), to yield chromatin fragments of roughly 200–500 bp in length (as determined by sonication optimization test experiments). At this stage, a fraction of each sample was kept aside as input control. For immunoprecipitation, a portion of the sheared chromatin sample was diluted tenfold with dilution buffer and then immunoprecipitated via 2 h incubation with Dynabeads Protein A/G pre-coupled to either rabbit anti-acetyl-Histone H3 antibodies (AcH3, 2.5 μg per sample, Merck Millipore) or normal IgG. Lysine 14 acetylation is a marker of genes being actively transcribed into RNA, thus immunoprecipitation with anti-AcH3 antibodies allows an assessment of the relative activity at a promoter of interest. Following extensive washing with immunoprecipitation buffers 1 and 2, cross-linking was reversed via heat and reverse crosslinking buffer. DNA was purified from the samples by DNA purification magnetic beads before elution in DNA elution buffer. The relative association of AcH3 to the Nrf2 or β actin promoters was then quantitatively assessed in input control and immunoprecipitated samples via real-time qPCR (as described above) using validated primers directed against the mouse Nrf2 and β-actin promoter regions (for sequences see Supplementary Table 1). In all instances, samples were normalized to their respective non-immunoprecipitated input controls.

### Fluorescent-activated cell sorting

All procedures were authorized under a UK Home Office approved project licence and adhered to regulations specified in the Animals (Scientific Procedures) Act (1986). Cortical cells obtained from 12- to 14-week-old adult CD1 male mice were processed essentially as described in ref. 20. Briefly, mice were culled via decapitation, cortices quickly removed in Hibernate A (Hib A, Life Technologies Ltd) and then minced on ice before incubation in Accutase (Merck Millipore) for 30 min at 4 °C on a rotator. After centrifugation, cortices were resuspended in Hib A and homogenized with a fire-polished Pasteur pipette. Debris was removed via filtration through a 40 μM filter and three-step Percoll gradient. Cell pellets were resuspended in Hib A and an equal amount of cold absolute ethanol was added. Cells were then left on ice for 15 min with occasional inversion, before centrifugation and resuspension in RNAse/DNAse-free PBS and incubation with an anti-NeuN primary antibody (6:1,000, Merck Millipore) for 30 min at 4 °C on a rotator. Cells were washed, resuspended in RNAse/DNAse-free PBS containing Cy3-donkey anti-mouse secondary antibody and left to rotate for 15 min at 4 °C (1:200, Stratech Scientific Limited), before washing and resuspension in RNAse/DNAse-free PBS. Cells were briefly incubated with DNA-binding dye 7AAD (Life Technologies Ltd) to identify nucleated events and facilitate gating and sort parameters, before sorting using a FACS-Jazz cell sorter (BD Biosciences). All non-7AAD^+^ events were omitted as they were considered non-nuclear. To establish the normal light scattering characteristics of the gated population (7AAD^+^ events), a sample that had not been exposed to antibodies was first sorted. Following this a sample that had only been exposed to the Cy-3-conjugated secondary antibody was sorted to establish the maximum threshold for nonspecific binding. Following establishment of the appropriate gating thresholds, experimental samples were sorted into two tubes based on their emission profile as either NeuN^+^/7AAD^+^ or NeuN^−^/7AAD^+^ (see Supplementary Fig. 1e). A total of 300 K NeuN^+^ and 300 K NeuN^−^ cells were collected per repeat. Purity testing was done for each n to ensure sorting accuracy, sorted samples consistently showed >98% purity profiles (see Supplementary Fig 1e). RNA was immediately isolated as describe above using the Roche High-Pure RNA Isolation Kit. Following RNA isolation and cDNA synthesis, expression of cell-specific gene markers was assessed by qRT–PCR to confirm enrichment of neuronal and non-neuronal genes in appropriate NeuN^+^ and NeuN^−^ FACS populations.

### Reporter assays

A firefly luciferase-based reporter gene construct (M50 Super 8x TOPFlash, a Beta-catenin reporter with TCF/LEF sites upstream of a luciferase reporter) was transfected along with a renilla expression vector (pTK-RL), and where relevant, other expression vectors of interest on DIV8. Neurons were stimulated with H_2_O_2_ 48 h after transfection (for 16 h). Luciferase assays were performed using the Dual Glo assay kit (Promega) with Firefly luciferase-based reporter gene activity normalized to the Renilla control (pTK-RL plasmid) in all cases. All luminescent assays were performed on a FLUOstar OPTIMA (BMG Labtech). Light collection time and gain were set such that counts were substantially lower than the maximum level collectable.

### Immunofluorescence

Cells were fixed with formaldehyde, washed with PBS, permeabilized with PBS+NP-40 (Life Technologies Ltd) and washed again before overnight rotating incubation with primary antibodies diluted in PBS at 4 °C. The following day, cells were washed and antibody binding visualized via biotinylated secondary antibody/fluorophore-conjugated streptavidin. Where required, nuclei were counter-stained with DAPI. In all instances, non-saturating images were captured on a Leica AF6000 LX imaging system. Employed primary antibodies include: rabbit anti-Hmox1 (1:1,000, Stressgen Biotechnologies), mouse anti-GFAP (1:400, Sigma), rabbit anti-GFP (1:750, Life Technologies Ltd), Milli-Mark neuronal antibody cocktail (Millipore, 1:500) and Anti-Nrf2 (Cell Signaling). To measure Hmox1 immunofluorescence intensity, non-saturating images at × 20 were captured using the Leica AF6000 LX imaging system with DFC350 FX digital camera. Fluorescence intensity was quantified using Image J software. Fluorescence intensity was quantified for each cell across four or five fields, with each field containing 10–18 cells. To ensure consistency, settings were maintained across all conditions in a given experiment. Background intensity was subtracted from cellular intensity, and resulting values of approximately 70 cells per condition were averaged and normalized to control.

### Western blotting

For protein extraction experiments, cells were cultured in 35 mm dishes. To minimize the chance of post-translational modifications during the harvesting process, cells were immediately lysed in × 1.5 sample buffer (1.5 M Tris (pH 6.8); 15% glycerol; 3% SDS; 7.5% β-mercaptoethanol; bromophenol blue 0.0375%) and boiled at 100 °C for 10 min. Approximately 30 μg of protein was loaded per lane and the gel then subjected to gel electrophoresis and western blotting using the Xcell Surelock system with precast gradient gels (4–20%, Life Technologies Ltd), according to the manufacturer’s instructions. Gels were blotted onto polyvinylidene difluoride membranes, and blocked for 1 h at room temperature with 5% (w/v) non-fat dried milk in TBS with 0.1% Tween-20. Membranes were incubated overnight at 4 °C with primary antibodies diluted in the blocking solution: Anti-cJun (1:1,000, Cell Signalling), Anti-pSer73-cJun (1:1,000, Cell Signalling), Anti-Nrf2 (1:1,000, Cell Signaling), anti-β-actin (1:2,000, Sigma). For visualization of western blots, HRP-based secondary antibodies were used followed by chemiluminescent detection on Kodak X-Omat film. Appropriate exposures were taken such that bands were not saturated. Where required a stripping agent was utilized to enable detection of the same blot with a second antibody, as achieved through the use of Re-Blot Plus Stripping Solution (Millipore). Western blots were analysed by digitally scanning the blots, followed by densitometric analysis (ImageJ). Signals were normalized to either amounts of p-c-Jun relative to cJun, or to amounts of Nrf2 relative to β-actin as loading control. For figure preparation of example western blots, linear adjustment of brightness/contrast was applied (Photoshop) equally across the entire image, taking care to maintain some background intensity.

### Statistical analysis

Paired *t*-tests were used to compare non-independent data pairs, whereas Student’s *t*-tests were utilized when the two groups were not related. For studies employing multiple testing, we used a one- or two-way analysis of variance followed by Tukey’s or Bonferroni’s *post-hoc* test as appropriate. For all tests significance was set at *P<0.05. Analysis of cell death, dendritic morphology and PSD-95 puncta density was performed blind to the mouse genotype or other experimental history (drug treatment, plasmid transfected and so on). Error bars represent standard error of the mean.

## Additional information

**How to cite this article:** Bell, K.F.S. *et al.* Neuronal development is promoted by weakened intrinsic antioxidant defences due to epigenetic repression of Nrf2. *Nat. Commun.* 6:7066 doi: 10.1038/ncomms8066 (2015).

## Supplementary Material

Supplementary Information Supplementary Figures 1-3 and Supplementary Table 1
