# Supplementary material for: Neuronal development is promoted by weakened intrinsic antioxidant defences due to epigenetic repression of Nrf2
Source: Nat Commun. 2015 May 13;6:7066. doi: 10.1038/ncomms8066 (PMC4441249; doi:10.1038/ncomms8066)
Supplement: Supplementary Information — Supplementary Figures 1-3 and Supplementary Table 1 [file ncomms8066-s1.pdf]

Supplementary Material

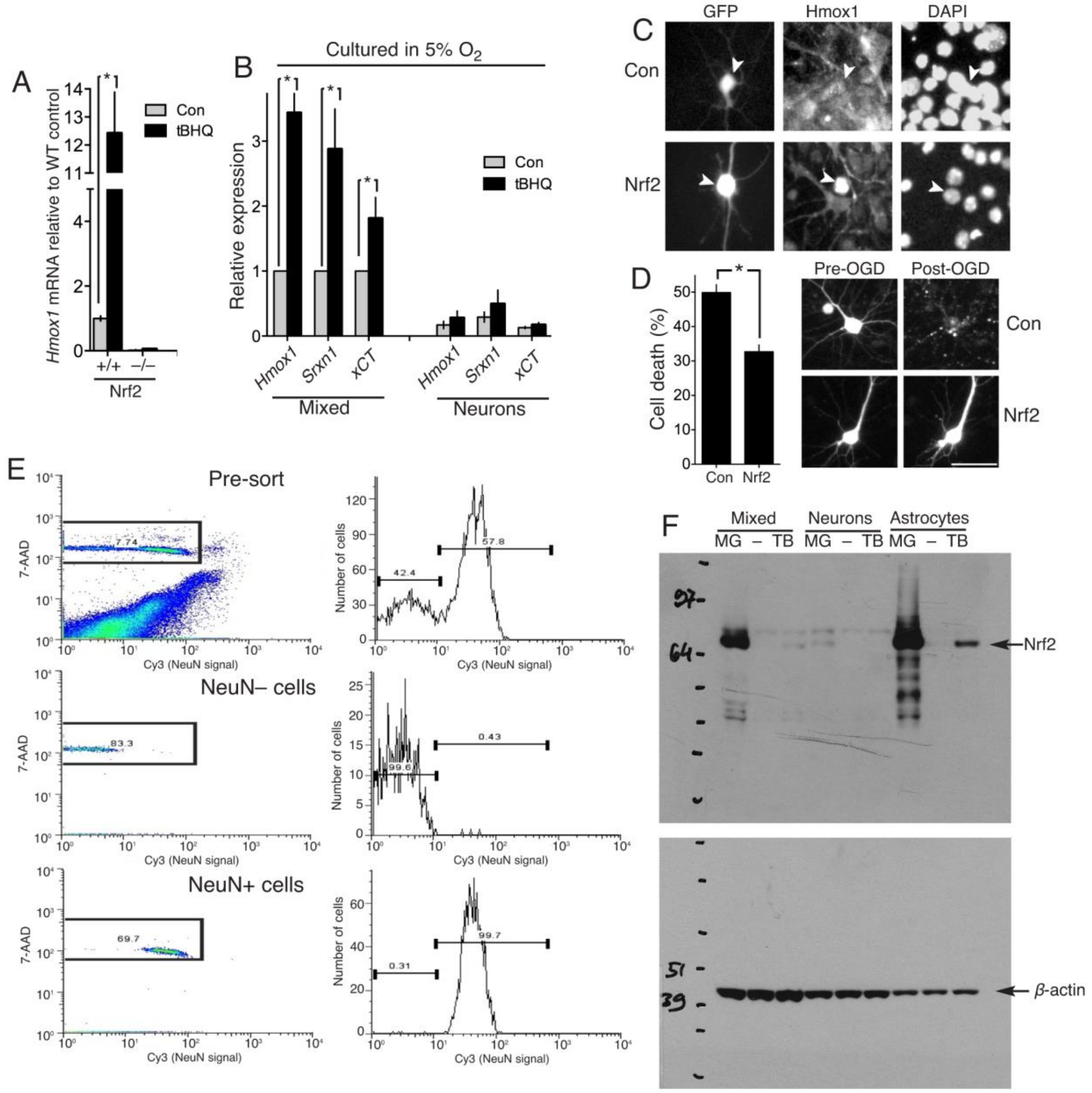

Supplementary Figure 1. (**A)** qRT-PCR demonstrating the Nrf2-dependent effect of Nrf2 activating drug tBHQ on mixed cortical cultures. tBHQ-dependent induction of Nrf2 target genes is only observed in wild type cells and is completely eliminated in the absence of Nrf2 (10 μM, 8h, *p<0.05, n=5-6). **(B)** Neurons cultures in 5% O2 conditions were treated with tBHQ (10μM, 8h) and qRT-PCR analysis of the indicated Nrf2 target genes analysed. *p<0.05 Student's *t*-test, (n=4)**.** **(C)** Immunofluorescent images demonstrating that the neuronal Nrf2 pathway is responsive if Nrf2 is present. Neurons were transfected with pNrf2 or control vector (β-globin) plus a eGFP co-transfection marker. 48h post-transfection the neurons were fixed and Hmox1 detected by immunofluorescence. Arrows indicate transfected cells. **(D)** Neurons were transfected with control vector or pNrf2, plus eGFP to monitor cell fate before and after (24 h later) 3h oxygen glucose deprivation (OGD). Example pictures (right) and quantitation (left) demonstrate a neuroprotective effect of Nrf2 expression in withstanding an OGD insult (*p<0.05, 25 cells/treatment, n=5, thus 125 cells quantified in total/transfection conditions). Scale bar = 30 μM. **(E)** FACS diagram illustrating the gating parameters utilised to FACS sort the adult mouse cortical homogenate based on 7-AAD positivity (indicative of a nucleated event) and NeuN immunofluorescence intensity. Note the presence of 2 distinct populations within the 7-AAD+ population, representing the neuronal (NeuN+) and non-neuronal (NeuN-) sub-populations (gating parameters employed here and through out, gated cells shown in rectangle). At right, representative images of purity tests illustrating the accuracy of the employed FACS gating parameters. In all purity tests sorting accuracy was greater than 98.5%. **(F)** Raw scanned films from Fig. 1k.

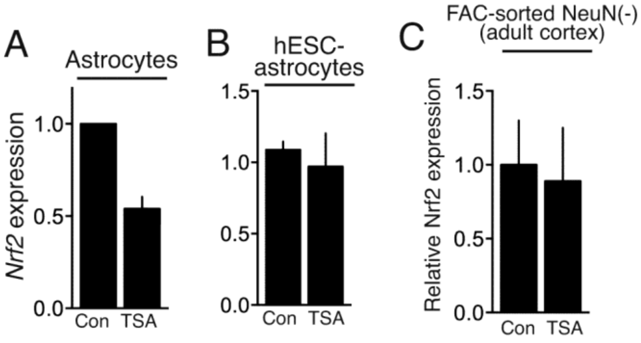

Supplementary Figure 2. Unlike in neurons, application of HDAC inhibitor TSA (2.5 μM, 8h) fails to increase *Nrf2* expression in **(A)** cortical mouse astrocytes (n=5) or **(B)** H9-hESC-derived astrocytes (n=5). **(C)** Intra-peritoneal injection of TSA (10mg/kg, 8h pre-FACS) in adult mice does not induce *Nrf2* expression in FACS sorted non-neuronal cells (NeuN-, n=4).

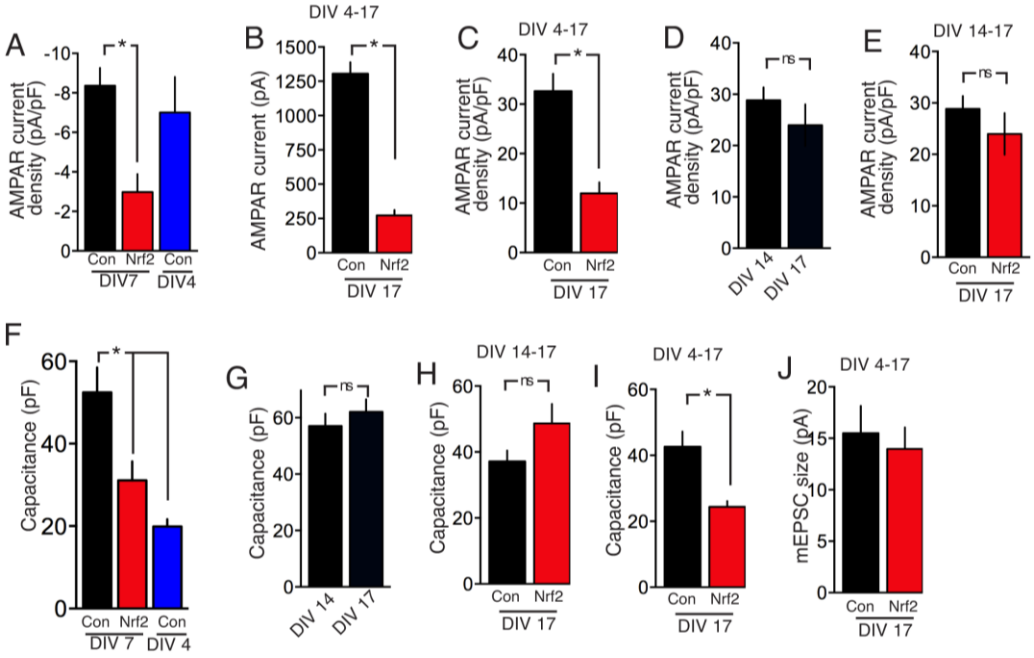

Supplementary Figure 3. (A) AMPA receptor current density is significantly reduced in Nrf2-expressing DIV 7 neurons (transfected on DIV 4), as compared to β-globin transfected controls *p<0.05, 2-way Anova plus Tukey's post-hoc test (n=8-11). **(B, C)** AMPA receptor current (B) and current density (C) is significantly reduced in Nrf2-expressing DIV 17 neurons when transfected on DIV 4, as compared to β-globin transfected controls (transfected at DIV 4) *p<0.05 (n=8 per condition). **(D)** AMPA receptor current density does not change significantly between DIV 14 and DIV 17 (n=10 (DIV 14), n=16 (DIV 17)). **(E)** AMPA receptor current density is not affected in Nrf2-expressing DIV 17 neurons when transfected on DIV 14 (n=12 per condition). **(F)** Neuronal membrane capacitance was measured in DIV 7 and DIV 4 neurons transfected as indicated. Capacitance increases strongly over the period DIV 4 to DIV 7, and this is attenuated by Nrf2 expression (*p<0.05, n=11, 10, 8). **(G)** Neuronal membrane capacitance does not change significantly between DIV 14 and DIV 17 (n=10 (DIV 14), n=16 (DIV 17)). **(H)** Neuronal membrane capacitance is not affected in Nrf2-expressing DIV 17 neurons when transfected on DIV 14 (n=12 per condition). **(I)** Neuronal membrane capacitance is significantly reduced in Nrf2-expressing DIV 17 neurons when transfected on DIV 4. *p<0.05, Student's t-test (n=8 per condition). **(J)** mEPSC size measured in neurons transfected as indicated on DIV4 and recorded on DIV17. *p<0.05 (n=9 control, n=8 Nrf2).

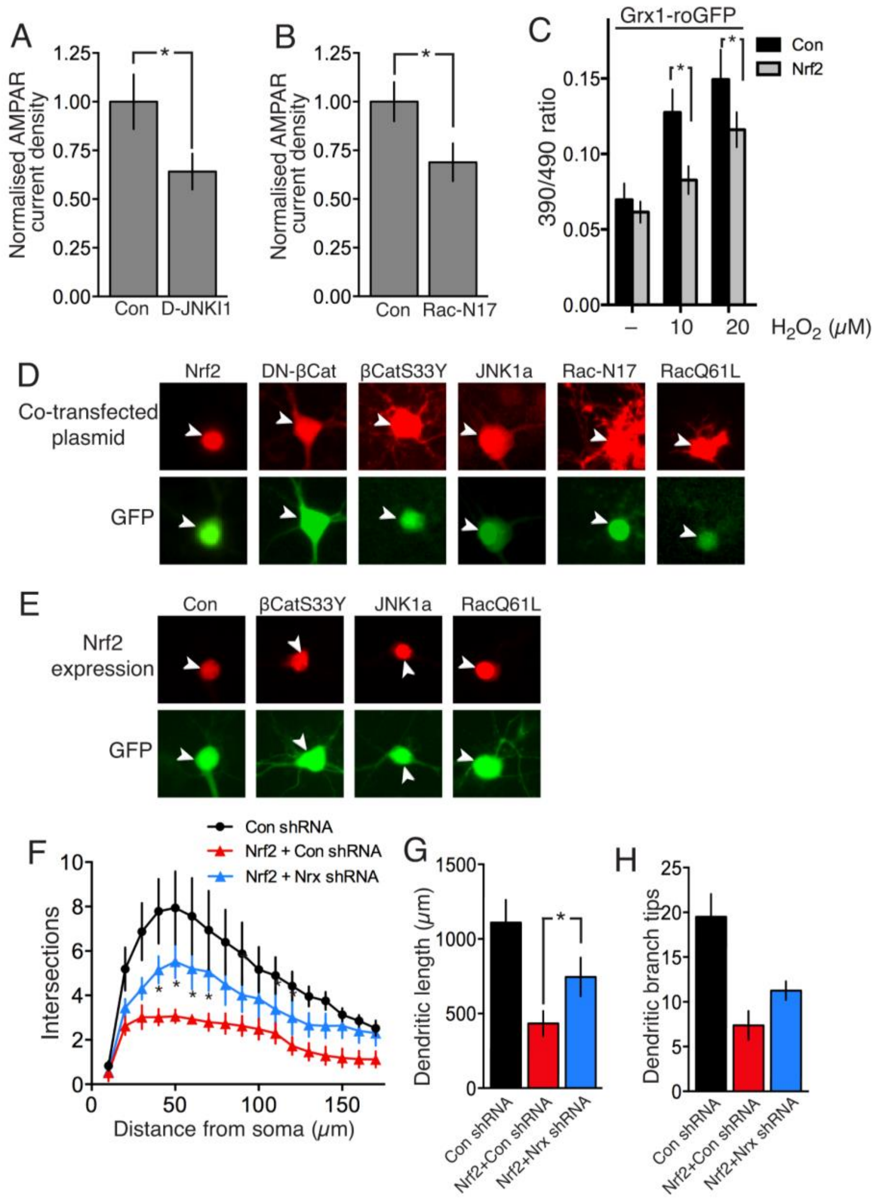

Supplementary Figure 4. (A) Neurons were treated with D-JNKI1 daily between DIV 4-6 where indicated, and AMPAR currents (normalized to capacitance) measured on DIV 7. Levels are expressed relative to vehicle-treated neurons. *p<0.05, Student's *t*-test (n=10 of each condition). **(B)** Neurons were transfected with Rac-N17 or control plasmids on DIV 4, and AMPAR currents (normalized to capacitance) measured on DIV 7. Levels are expressed relative to vehicle-treated neurons. *p<0.05, Student's *t*-test (n=15 of each condition). **(C)** Neurons expressing Grx1-roGFP plus either control (globin) or Nrf2-encoding plasmids were subject to live cell imaging during treatment with H_2O_2. Pairs of images were taken (ex 387±5 and 494±10; em 530±10 in both cases) and the ratio calculated. *p<0.05, 2-way Anova, plus Bonferronni's post-hoc test, n=6 independent experiments, total of 30 (Con) and 35 (Nrf2) transfected cells analysed. **(D)** To confirm expression of the plasmids used, neurons were transfected with peGFP plus the indicated plasmids and detected with the following antibodies (all at 1:250): Nrf2 (anti-Nrf2, Cell Signalling), DN-βCat-myc (anti-Myc, 9E10, Santa Cruz), βCatS33Y-Flag (anti-Flag, Cell Signalling), JNK-1a (anti-JNK, Novus Biologicals), RacN17-myc (anti-Myc, 9E10, Santa Cruz), RacQ61L-myc (anti-Myc, 9E10, Santa Cruz) Arrows point to the transfected cells. **(E)** Confirmation that Nrf2 expression is not affected under the experimental conditions described in Figs 4g-i, 4k-m and 4o-q. Nrf2 expression detected by immunofluorescence in cells co-transfected with peGFP plus the indicated 'rescue' plasmid-βCatS33Y, JNK1a or RacQ61L. F) Sholl analysis of neurons transfected as indicated. *p<0.05, 2-way Anova, plus Tukey's post-hoc test. * indicates a significant rescue, comparing the Nrf2+shRNA condition to Nrf2+Nrx shRNA. 48 cells analysed per condition within n=4 independent experiments. **(G, H)** Cells in (F) were analysed for dendritic length and terminal branch tip number.

Supplementary Table 1: QPCR primers

	Primer	FORWARD	REVERSE
**Mouse**	*Nrf2*	CAGCTCAAGGGCACAGTGC	GTGGCCCAAGTCTTGCTCC
	kNrf2-1 (FACS)	TTAAGCAGCATAGAGCAGGAC	GAACAGCGGTAGTATCAGCC
	Cat	GCGTCCAGTGCGCTGTAGA	TCAGGGTGGACGTCAGTGAA
	Gclc	CCAACCATCCGACCCTCTG	TGTTCTGGCAGTGTGAATCC
	Srxn1	GACGTCCTCTGGATCAAAG	GCAGGAATGGTCTCTCTCTG
	Hmox	AGCACAGGGTGACAGAAGAG	GGAGCGGTGTCTGGGATG
	xCT	ATACTCCAGAACACGGGCAG	AGTTCCACCCAGACTCGAAC
	Gapdh	GGGTGTGAACCACGAGAAT	CCTTCCACAATGCCAAAGTT
	18s	GTGGAGCGATTTGTCTGGTT	CAAGCTTATGACCCGCACTT
	Mal	CTTCACCACCTTCCCTGAC	TTCCAGAACTGAGGCACTG
	Grin1	CTGCGACCCCAAGATTGTCAA	TATTGGCCTGGTTTACTGCCT
	Gfap	ACAACTTTGCACAGGACCTC	CTGTGAGGTCTGGCTTG
	Rpl13a	GAGGTCGGGTGGAAGTACCA	TGCATCTTGGCCTTTTCCTT
**Human**	GRIN1	AGGAACCCCTCGGACAAGTT	CCGCACTCTCGTAGTTGTG
	NFE2L2	TGATTGACATACTTTGGAGGC	TCTTCATCTAGTTGTAACTGAGCG
	18s	GTGGAGCGATTTGTCTGGTT	CAAGCTTATGACCCGCACTT
**ChIP (mouse)**	Nrf2 Promoter	GAGGTCACCACAACACGAAC	ATCTCATAAGGCCCCACC C
	ß-Actin Promoter	CCGGTCGAGTCGCGTCCACC	GGCGAACTGGTGGCGGGTGT
